# Chronically Stable, High‐Resolution Micro‐Electrocorticographic Brain‐Computer Interfaces for Real‐Time Motor Decoding

**DOI:** 10.1002/advs.202506663

**Published:** 2025-09-06

**Authors:** Erda Zhou, Xiner Wang, Jizhi Liang, Yang Liu, Qinrong Yang, Xingchen Ran, Lei Xia, Xiang Zou, Changjiang Liu, Liuyang Sun, Lei Peng, Liang Chen, Ying Mao, Zehan Wu, Tiger H. Tao, Zhitao Zhou

**Affiliations:** ^1^ 2020 X‐Lab Shanghai Institute of Microsystem and Information Technology Chinese Academy of Sciences Shanghai 200050 China; ^2^ School of Graduate Study University of Chinese Academy of Sciences Beijing 100049 China; ^3^ Neuroxess Co., Ltd. Shanghai 200023 China; ^4^ Department of Neurosurgery Huashan Hospital of Fudan University Shanghai 200040 China; ^5^ School of Integrated Circuits University of Chinese Academy of Sciences Beijing 100049 China; ^6^ Guangdong Institute of Intelligence Science and Technology Zhuhai Guangdong 519031 China; ^7^ Tianqiao and Chrissy Chen Institute for Translational Research Shanghai China; ^8^ State Key Laboratory of Transducer Technology Shanghai Institute of Microsystem and Information Technology Chinese Academy of Sciences Shanghai 200050 China

**Keywords:** brain‐computer interfaces, flexible conformal micro‐electro‐mechanical systems, high‐resolution micro‐electrocorticography, real‐time motor decoding

## Abstract

Brain‐computer interfaces (BCIs) enable communication between individuals and computers or other assistive devices by decoding brain activity, thereby reconstructing speech and motor functions for patients with neurological disorders. This study presents a high‐resolution micro‐electrocorticography (µECoG) BCI based on a flexible, high‐density µECoG electrode array, capable of chronically stable and real‐time motor decoding. Leveraging micro‐nano manufacturing technology, the µECoG BCI achieves a 64‐fold increase in electrode density compared to conventional clinical electrode arrays, enhancing spatial resolution while featuring scalability. Over a 203‐day in vivo experiment, high‐resolution µECoG carrying fine spatial specificity information demonstrated the potential to improve decoding performance while reduce implanted devices size. These advancements provide a pathway to overcome the limitations of conventional ECoG BCIs. During awake surgery, the µECoG BCI enabled game control after 7 min of model training. Furthermore, during practice of 19.87 h, the participant achieved cursor control with a bit rate of 1.13 bits per second (BPS) under full volitional control, and the bit rate reached up to 4.15 BPS with enhanced user interface. These results show that the µECoG BCI achieves comparable performance to intracortical electroencephalographic (iEEG) BCIs without intracortical invasiveness, marking a breakthrough in the clinical feasibility of flexible BCIs.

## Graphical abstract

A high‐resolution micro‐electrocorticographic (µECoG) brain‐computer interface (BCI) for real‐time motor decoding is reported. The application of flexible, scalable µECoG electrode arrays overcomes the insufficient spatial resolution in conventional ECoG BCIs. The real‐time motor and motor imagery decoding achieved in the long‐term in vivo experiment and clinical practices demonstrates the practical value of flexible µECoG BCIs.

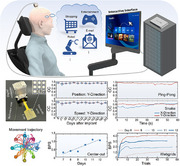

## 1 Introduction

Motor impairments caused by neurological disorders such as stroke, spinal cord injury, and amyotrophic lateral sclerosis severely limit patients’ independence and mobility, which drastically affects their quality of life and potentially shortens their life expectancy. While some computer‐aided technologies offer partial improvements, they are often inefficient and difficult to use, providing a less‐than‐satisfactory experience.^[^ ^1^, ^2^ ^]^ BCIs allow the patients to interact with assistive devices directly by their brain activity, offering more efficient approaches to reconstruct motor function and regain basic self‐care abilities in daily lives.^[^ ^3^, ^4^, ^5^ ^]^

The overall performance of BCIs critically depends1 on the acquisition methods employed for brain signals, such as electroencephalography (EEG), iEEG, and ECoG. EEG, while non‐invasive, exhibits signal attenuation across the cerebrospinal fluid, dura, skull, and scalp.^[^ ^6^, ^7^ ^]^ Due to suboptimal signal quality (signal‐to‐noise ratio, SNR) and limited spatiotemporal resolution, EEG BCIs are typically restricted to simple decoding tasks and suffer from high latency.^[^ ^8^, ^9^, ^10^ ^]^ In contrast, iEEG acquired via intracortical microelectrode arrays (iMEAs) achieves excellent spatial (single‐neuron) and temporal (sub‐millisecond) resolution, but is constrained by intracortical invasive trauma and limited brain coverage.^[^ ^11^, ^12^, ^13^, ^14^ ^]^ ECoG recorded by electrode arrays placed on the cerebral cortex achieves a balance between signal quality and invasiveness, which has shown great potential for motor and motor imagery decoding (Table S1, Supporting Information).^[^ ^15^, ^16^, ^17^, ^18^, ^19^, ^20^ ^]^ Moreover, its broader brain coverage compared to iMEAs, enables advancements in decoding complex human behaviors, such as speech decoding.^[^ ^21^, ^22^ ^]^

However, the electrode density and the throughput of conventional clinical ECoG electrode arrays are inherently limited in scalability due to manufacturing constraints, potentially impeding neural decoding advancements. Additionally, device miniaturization difficulties necessitate larger craniotomies, exacerbating surgical trauma. Recently, high‐throughput and high‐density µECoG electrode arrays fabricated leveraging micro‐nano manufacturing technology have demonstrated considerable promise in addressing these challenges.^[^ ^23^, ^24^ ^]^ Nevertheless, chronic in vivo validation and clinical implementation of flexible µECoG arrays for motor or motor imagery decoding remain scarce, and the interdependence among electrode density, brain coverage, and decoding performance requires systematic investigation.

In this study, we developed a high‐resolution µECoG BCI, leveraging a flexible µECoG electrode array with a density of 64 electrodes/cm^2^. The array's ultrathin, mesh‐configured recording area enables conformal cortical adhesion (Figure S1, Supporting Information), while thickened lead zones provide sufficient mechanical robustness for chronic, stable acquisition of high‐fidelity neural signals. Over a 203‐day in vivo experiment, real‐time three‐dimensional motor decoding with the mean accuracy of 0.84 was achieved in a Labrador dog. We demonstrated that increased electrode density enhances decoding performance without expanding brain coverage, underscoring the significance of high‐resolution µECoG for advanced BCI control. Clinical intraoperative motor decoding and short‐term motor imagery experiments were subsequently conducted. Following 7 min of data collection and decoder training, the participant successfully operated Ping‐pong and Snake games via brain activity during awake surgery. In a 12‐day in vivo experiment, bit rates during Center‐out and Webgrids paradigms progressively increased with trial accumulation, reaching iEEG BCI‐comparable levels within reduced practice duration.^[^ ^14^ ^]^ These results provide evidence that the clinical feasibility of leveraging high‐resolution µECoG BCIs with neural plasticity to achieve chronically stable motor function reconstruction.

## 2 Results

**Figure 1:**
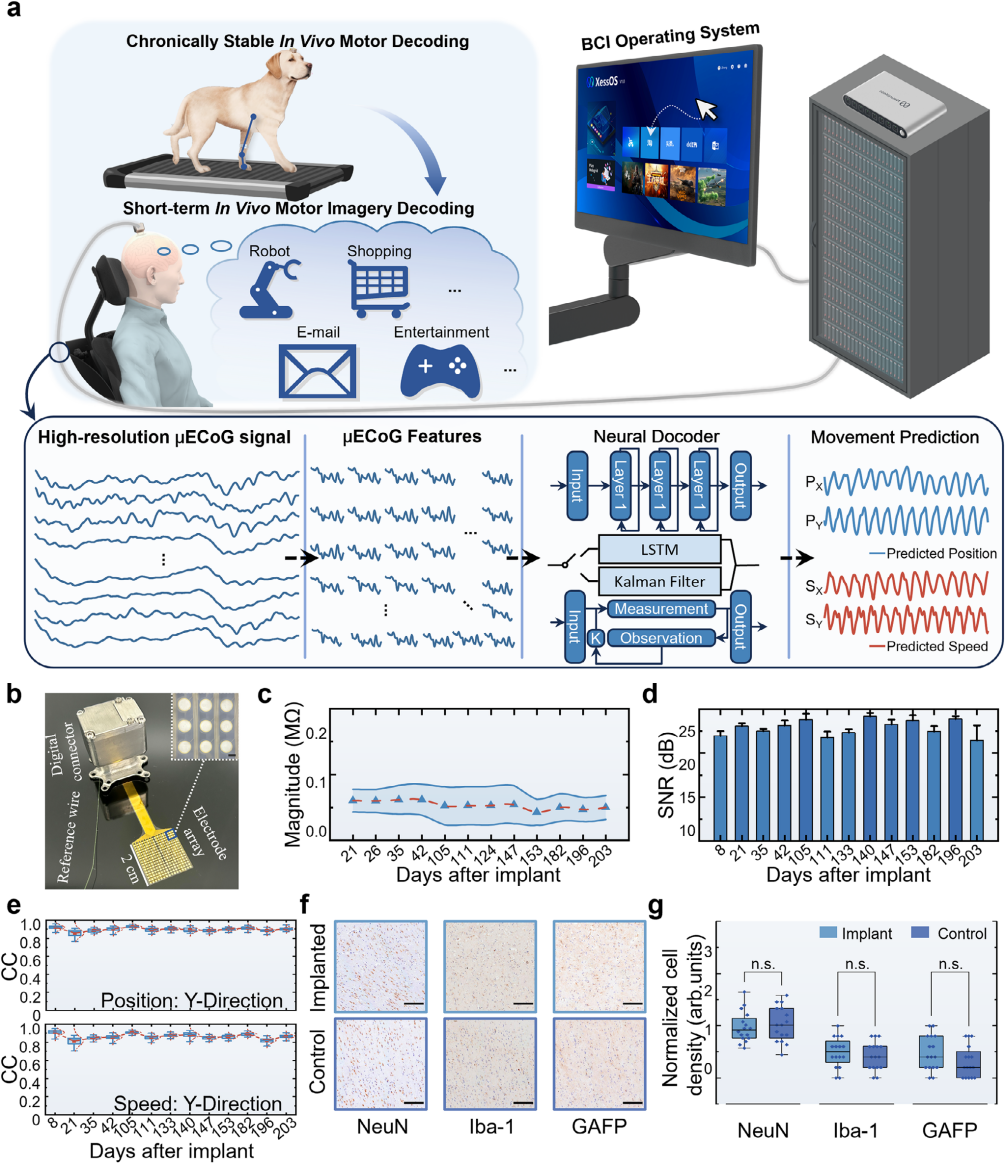
Overview and long‐term performance of the high‐resolution µECoG BCIs. a) The schematic and workflow of the real‐time motor decoding and motor imagery decoding based on the µECoG BCIs. b) The integrated implant (the scale bar is 1 cm) for the µECoG BCIs. The inset displays zoom‐in of the geometric structure of the µECoG electrode arrays (the scale bar is 500 µm). c) Electrode impedances during the long‐term experiment lasting 7 months. The blue triangle markers and the red dashed line indicate the mean values of each session and the blue shadow area indicate the standard deviations. d) SNR of µECoG signals in each session during the long‐term experiment. e) Decoding accuracies (Correlation coefficient, CC) for all sessions in the Y‐Direction. For each session, the box plot represents quartiles range with medians (blue lines) and mean values (square red markers) labeling and the whiskers denote maximum and minimum values. The fitted normal distribution of in‐session decoding accuracy is shown to the right of each box. f) Immunohistochemical staining was performed on tissue sections of the implanted electrode site and the control site (mirror symmetry with the implanted electrode site according to the mid‐sagittal plane) after the in vivo experiment (the scale bars are 100 µm). g) The results of cell counting (the data were normalized to those on the control site) and statistical analysis (for the data followed a normal distribution, two‐sided paired t‐tests were applied, otherwise Wilcoxon signed‐rank tests were conducted for significance. ns, *p* > 0.05, n = 16) of neurons (NeuN), microglia (Iba‐1), and astrocytes (GAFP).

The workflow of the high‐resolution µECoG BCI we proposed is outlined in **Figure** **1**a. During each session, high‐spatiotemporal‐resolution µECoG signals are acquired by an integrated implant (Figure 1b; Figure S2, Supporting Information). Subsequently, the signals undergo preprocessing where the power spectral density (PSD) of the high‐gamma (HG) band is extracted as feature vectors per electrode channel. Long short‐term memory (LSTM)‐based neural decoder and position‐velocity Kalman filter decoder (Figure S3, Supporting Information) are designed to predict actual or imagined movements from the PSD feature matrix, generating movement trajectories for motion synthesis or cursor control. Additionally, an interactive interface provides visual feedback and enables application functionalities. The system implements a cloud‐edge‐end architecture, allocating pre‐processing of raw data to edge devices, thereby reserving high‐performance servers for model training and neural decoding computations. For cross‐session applications, real‐time motor decoding was achievable after 5‐7 min of recalibration, demonstrating operational efficiency. For motor imagery decoding, a progressive calibration protocol enabled participants to attain full volitional cursor control within ≈30 min.

The µECoG electrode array, serving as the core component of the BCI system, features an electrode diameter of 850 µm and inter‐electrode spacing of 1250 µm. Pre‐implantation characterization revealed >95% of electrodes exhibiting low impedance (<1 MΩ at 1 kHz) and root‐mean‐square (RMS) noise <2 µV in phosphate‐buffered saline (Figure S4, Supporting Information), enabling high‐fidelity ECoG signal acquisition for volumetric neural data generation. Subsequently, a signal processing unit is connected to the electrode array through a flexible printed circuit (FPC), and a customized titanium enclosure is used as watertight and airtight encapsulation to enhance the robustness for long‐term operation. Existing micro‐electro‐mechanical systems (MEMS)‐fabricated high‐density µECoG arrays lack comprehensive assessment of chronic in vivo decoding stability, as electrode performance, algorithmic adaptability, and device‐induced inflammatory responses collectively determine BCI robustness for high‐accuracy real‐time motor decoding. To address this gap, we conducted real‐time motor decoding over 203 days in a Labrador dog. The experimental results revealed impressive stability with only a 5.49% decrease in yield of µECoG electrodes (Figure 1c; Figure S5, Supporting Information). For each session, we calculated the PSD and SNR to evaluate the signal quality. The frequency characteristics of signals from most electrode channels remain consistent across sessions (Figure S6, Supporting Information), and the SNR stabilized at >20 dB (Figure 1d), demonstrating the chronical stability of the µECoG BCIs. By comparing the predicted and real trajectories of each session, it is found that the µECoG BCI accurately captured and tracked the trends of kinematic features, and this capability was maintained throughout the entire in vivo experiment period (Figures S7 and S8, Supporting Information).

To quantitatively evaluate decoding performance, we calculated Pearson correlation coefficients between the predictions and the real recordings, providing a measure of motor decoding accuracy within each session (Figure S9, Supporting Information). Longitudinal analysis over 203 days demonstrated consistently high accuracy: 0.87 in the X‐direction, 0.90 in the Y‐direction and 0.83 in the Z‐direction for position, and 0.82 in the X‐direction, 0.86 in the Y‐direction and 0.78 in the Z‐direction for speed. In addition, the performance shows high stability, as reflected in the mean standard deviation: 0.03 in the X‐direction, 0.02 in the Y‐direction and 0.06 in the Z‐direction for position, and 0.04 in the X‐direction, 0.03 in the Y‐direction and 0.06 in the Z‐direction for speed (Figure 1e; Figure S10, Supporting Information). Notably, the Y‐direction ‐ corresponding to primary knee flexion kinematics ‐ exhibited superior performance attributable to greater movement variance magnitude Following the in vivo experiment, an immunohistochemical analysis was conducted. Histopathological analysis (Figure 1f; Figure S11, Supporting Information) quantitatively confirmed no evidence of neuronal loss between µECoG‐implanted regions and contralateral homotopic controls (mid‐sagittal plane symmetry reference). Quantitative analysis confirmed no significant astrocyte/microglia proliferation ‐ key inflammatory markers ‐ further validating the system's biosafety (Figure 1g). These collective findings substantiate the translational potential of µECoG BCIs.

**Figure 2:**
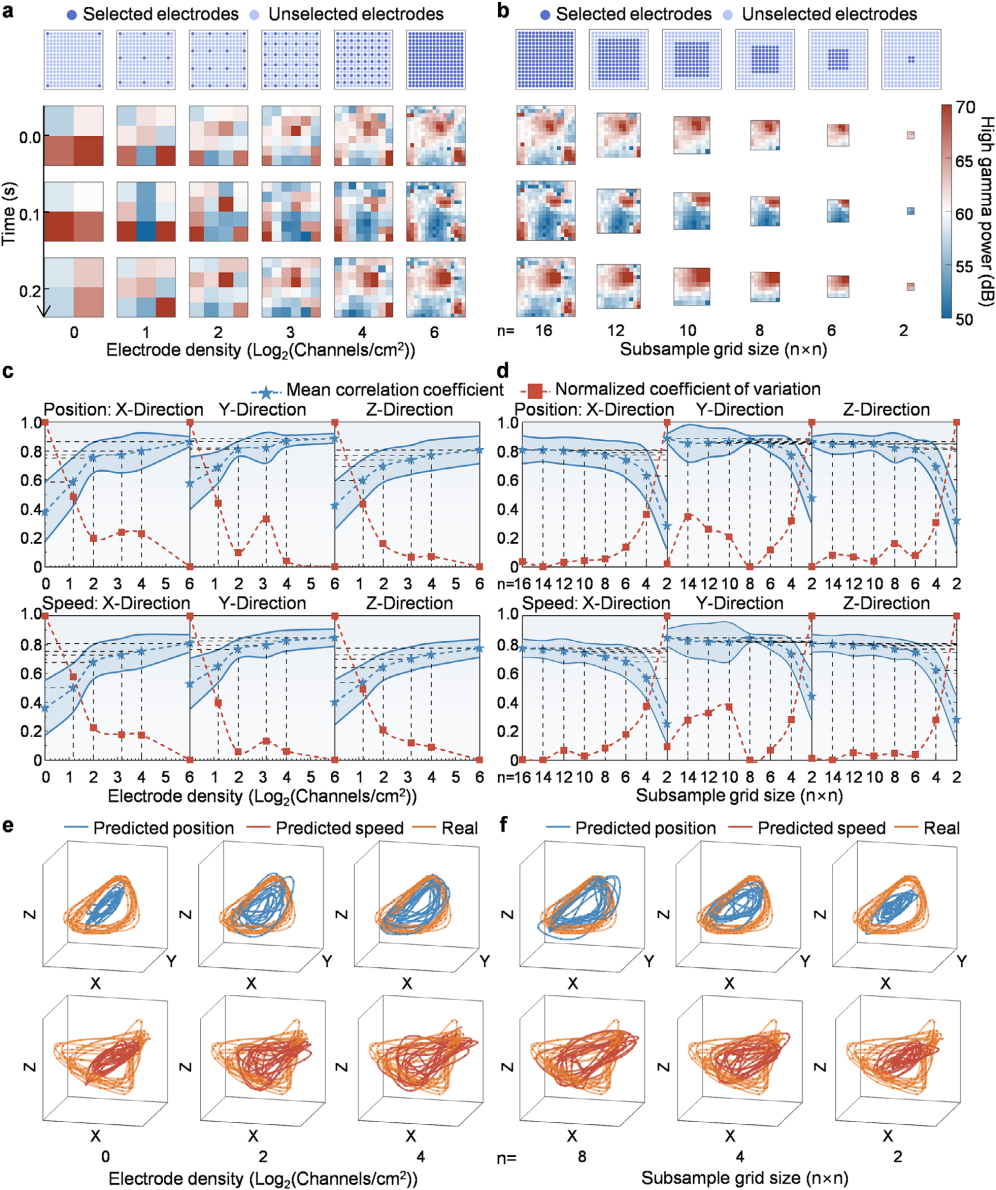
Improvement of motor decoding performance via increasing electrode density. a) The spatial distribution of HG power (the mean value of the 70∼150 Hz frequency band signal within a 100 ms time bin) for µECoG signals recorded by subsets with increasing electrode density. The spatial resolution is obviously improved. b) The electrode density is kept constant while the subsample grid size is gradually reduced to the center of the µECoG electrode array to simulate the reduction of brain coverage. The calculation of HG power is same as (a). c), d) Influence of increasing electrode density (a) and reducing subsample grid size (b) on decoding accuracy and variability. The blue pentagram markers indicate mean values for decoding accuracy, shadow areas show standard deviations, and red square markers denote normalized coefficients of variation. e), f) Examples of real and predicted trajectories of kinematic features (position and speed) decoded using electrode subsets with same throughput but different densities (c) and subsample grid size (d).

Previous studies have demonstrated that ECoG signals exhibit spatially constrained propagation with complex spatiotemporal dynamics, particularly in the 75‐300 Hz frequency band, necessitating submillimeter‐scale spatial sampling.^[^ ^25^, ^26^ ^]^ While high‐resolution µECoG signals have been proven to significantly improve speech decoding performance, few in‐depth studies on motor decoding have been carried out.^[^ ^27^ ^]^ Therefore, elucidating the relationship between electrode density and motor decoding performance is critical for optimizing µECoG array design. In a 2 cm × 2 cm area, we gradually increased the electrode density and calculated the HG power of the signal from each electrode. As electrode density increased, neural activity was represented with greater resolution, enabling more precise identification of cortical regions associated with movement (**Figure** **2**a). These movement‐related cortical areas are easily overlooked by electrode arrays with centimeter‐scale density because they are may be located between adjacent electrodes. Consequently, the neural activity in these regions may be aliased with surrounding irrelevant signals, resulting in the loss of important information. Prior to the implantation, key brain regions are usually identified by methods such as functional magnetic resonance imaging (fMRI) to facilitate the attachment of the µECoG electrode array in the correct location. With a constant electrode density, there is a choice to expand the brain coverage to obtain as much information as possible. However, the increase in throughput that accompanies the expansion could increase the time cost of signal processing, causing difficulties in device‐to‐device communication and real‐time decoding. Moreover, minimizing coverage reduces craniotomy extent and surgical trauma. Therefore, it is worth examining whether high‐resolution µECoG signals can be decoded efficiently into kinematic features without a substantial increase in throughput. In order to eliminate the effects introduced by the implanted region, subsets of electrode channels with varying grid sizes that gradually contract toward the center of the µECoG electrode array are selected to explore this issue (Figure 2b).

To evaluate electrode density effects on motor decoding performance, uniformly distributed electrode subsets of varying densities were analyzed within a fixed cortical area. These subsets were applied for decoding to assess the accuracy and variability (Figure 2c). Macroscopically, as electrode density increased, the decoding accuracy improved and variability decreased, both at a gradually diminishing rate, suggesting a potential trade‐off in enhancing stability through further density increases. The decoding accuracies in three directions all reached their maximal value at an electrode density of 64 channels/cm^2^. Subsequently, to identify the inflection point where decoding accuracies start to plummet, brain coverage and throughput were incrementally reduced. Decoding accuracies exhibited a gradual decline with diminishing coverage and transitioned to a sharp decrease at a subsample grid size of 6×6 (Figure 2d). Collectively, these findings demonstrate that high‐resolution µECoG enables optimal performance without extensive cortical coverage, thereby supporting craniotomy minimization through micro‐scale, high‐density electrode arrays. Notably, Y‐direction coefficient of variation fluctuations during this process may originate from contamination by movement‐unrelated neural signals in the decoding pipeline.

To gain a more intuitive understanding of the improvements in decoding performance, the predicted trajectories in three‐dimensional space, alongside the real trajectories were plotted (Figure 2e,f; Figures S12 and S13, Supporting Information). The enhancement in decoding performance improved the consistency between the predicted and real movements. In applications involving motion synthesis or prosthetic control, this may improve users’ proprioceptive feedback, enhancing ease of use and overall experience. Furthermore, it has been found that the mechanisms behind performance improvements from increasing density and expanding brain coverage are different. Increasing electrode density facilitates the revelation of detailed information embedded within the spatial structure, and the correlations within the HG band of inter‐electrode signals exhibit a very gradual increase. However, the correlations increase rapidly as the subsample grid size is reduced, suggesting that expanding brain coverage may enhance the diversity of information obtained from other cortical regions (Figure S14, Supporting Information). To transcend current performance ceilings, the paramount challenge remains escalating electrode density without compromising signal quality, thereby resolving finer‐grained spatiotemporal dynamics of neural population activity.

**Figure 3:**
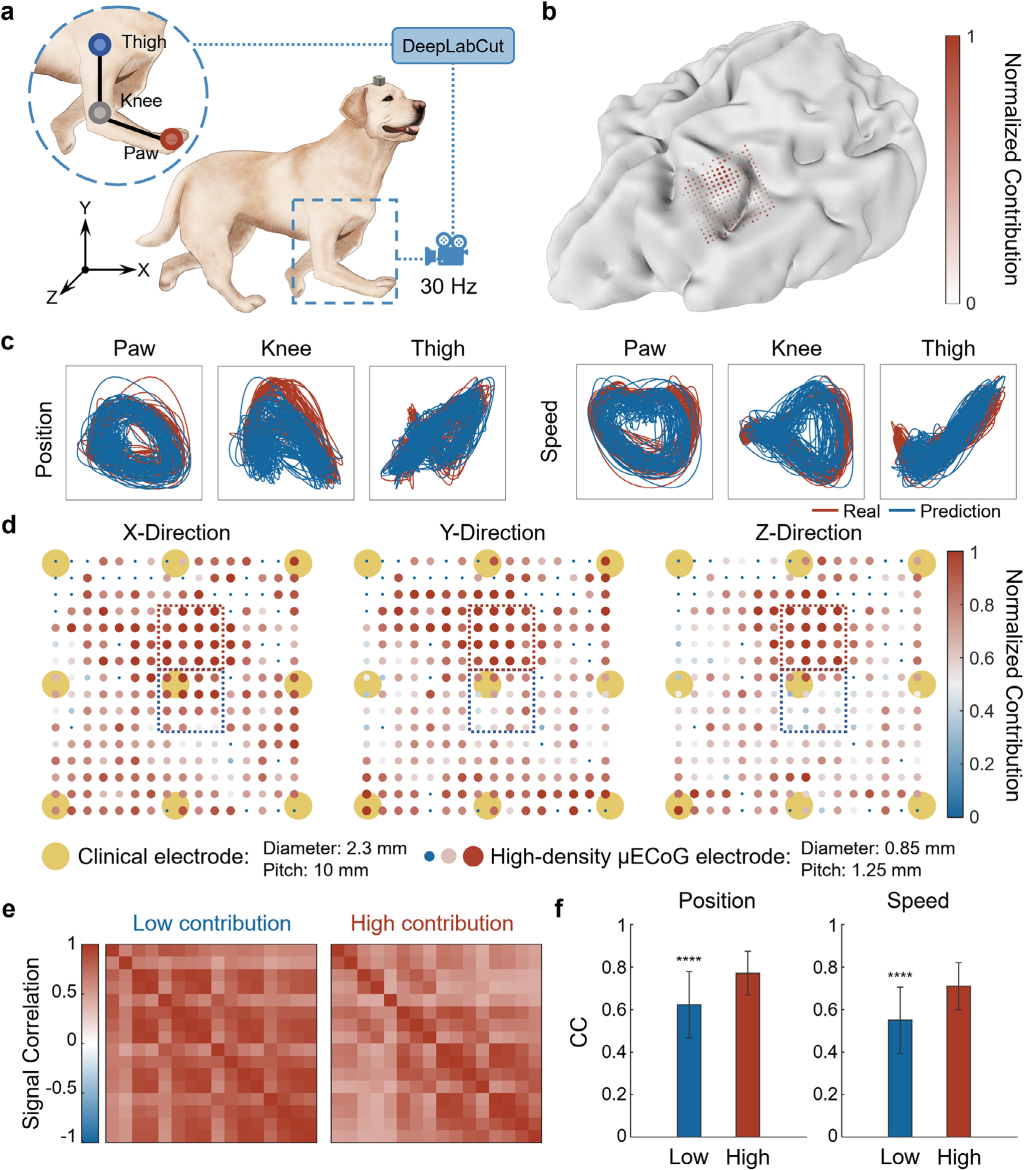
Multi‐joint motor decoding and the fine spatial structure of motor encoding. a) Definition of coordinate axes and acquisition of real movement trajectories of joints. b) Spatial distribution of normalized contributions of electrode channels to motor decoding on brain MRI reconstruction leveraging 3D Slicer.^[^ ^37^ ^]^ c) Examples of real versus predicted trajectories in multi‐joint motor decoding. d) Spatial distribution of normalized contributions to the motor decoding in three directions respectively. The encoding of movements in M1 exhibits spatial specificity on millimeter‐scale, and the fine spatial structure could be mapped via µECoG electrode arrays. The yellow circles represent conventional clinical electrodes. e) Signal correlation within HG band of inter‐electrode in electrode subsets with high and low contribution. f) µECoG signals acquired by high contribution subset electrodes exhibit significantly higher accuracy for instantaneous position and speed decoding (position decoding, *p* = 7.17e‐7, n = 39; speed decoding, *p* = 1.37e‐6, ****p < 0.0001, n = 39).

Current research on ECoG‐based BCIs for motor decoding typically focuses on collecting neural activity from the primary motor cortex (M1) to decode multi‐joint and multi‐directional movements. However, due to insufficient resolution, the consistency of the spatial distribution of neural activity encoding kinematic features across movements in different joints and directions remains unclear. Additionally, there is a lack of understanding regarding why high‐resolution µECoG improves decoding performance. Our results establish that the developed µECoG array enables high‐spatial‐resolution signal acquisition, providing a critical experimental platform to address these questions. To validate the functionality of the µECoG BCIs for multi‐joint motor decoding, the movement trajectories of the paw, knee, and thigh joints were concurrently recorded using computer vision‐based motion capture techniques (**Figure** **3**a). Before the implantation of the µECoG electrode array, the dog's brain was reconstructed using MRI, and the M1 was localized (Figure 3b). Following decoder training, all joint kinematics were simultaneously decoded from µECoG signals in real‐time (Figure 3c; Movie S1, Supporting Information). Subsequently, a random forest model was constructed to evaluate the importance of electrode channels, and logistic regression was employed to identify the subset of electrodes that provided optimal decoding performance. In each session, the reduction of Gini impurity caused by each electrode channel was used as a quantitative measure to assess the importance of electrode channels within the optimal subset, while others were deemed to contribute minimally to the decoding process. Despite M1's established motor role, intra‐M1 functional topography exhibits nuanced spatial segregation of movement‐encoding domains.

To investigate motor encoding in different directions, the previously mentioned method was employed to select the optimal subset in each session. The frequency with which each electrode appearing in the optimal subset was considered its contribution to long‐term decoding, and the normalized contribution was mapped onto the actual spatial distribution of the electrodes (Figure 3d). Crucially, electrodes contributing to all three directional decoders showed consistent spatial distributions and contribution magnitudes, indicating multidirectional movement encoding capacity within singular cortical domains. However, the number of electrodes contributing to decoding kinematic features in the X and Y directions was greater and more widely distributed compared to those in the Z direction. This discrepancy may be due to the larger amplitude of joint movement in the X and Y directions. To gain further insight, the normalized average electrode importances across sessions were plotted according to their spatial distributions (Figure S15, Supporting Information). Notably, the same electrode exhibited slight specificity in its importance for decoding different directions. Thus, while single cortical regions encode multidirectional movements, high‐spatial‐resolution µECoG resolves directionally tuned micro‐architectures.

High‐resolution sampling of ECoG can uncover the spatial specificity of the cortex, yet the advantages of this capability have not been fully elucidated. A subset of 4×4 with the highest average contribution across all directions was selected as the high‐contribution subset. Conversely, a neighboring subset of the same size with a lower average contribution was chosen as the low‐contribution subset. The signal correlations within the HG band for both subsets were calculated, and the results showed that the inter‐set correlation within the high‐contribution subset was lower than that of the low‐contribution subset, indicating that the high‐contribution subset captured more movement‐evoked neural activity, which remained consistent over the entire implantation period (Figure 3e; Figures S16 and S17, Supporting Information). Critically, the high‐contribution subset yielded significantly higher mean decoding accuracy across all three kinematic dimensions (Figure 3f; Figures S18 and S19, Supporting Information). These findings suggest that the high‐resolution µECoG's decoding enhancement may stem from the precise spatially specific neural activity in small, highly task‐related brain regions (Figures S20 and S21, Supporting Information).

**Figure 4:**
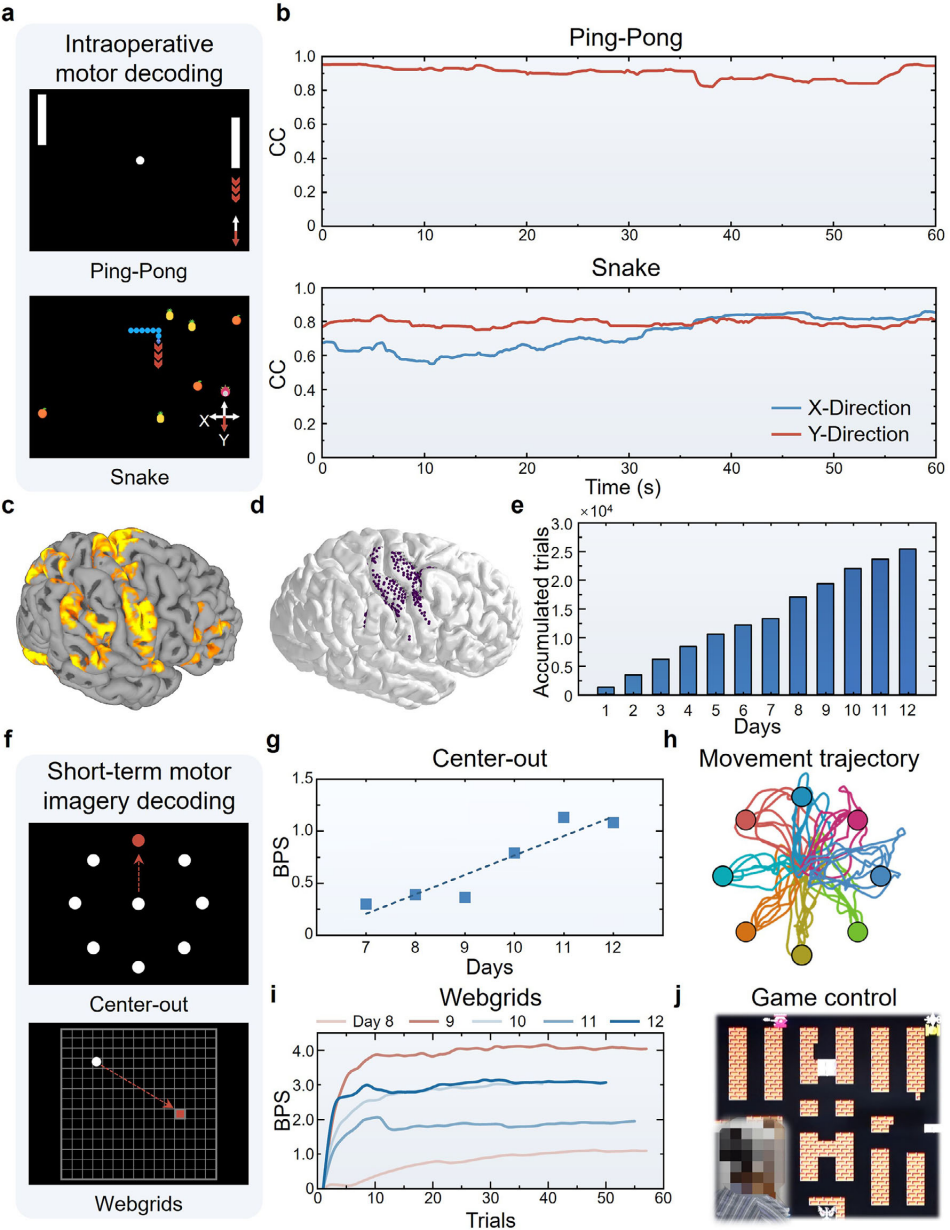
Clinical intraoperative control and short‐term stable control of the µECoG BCI for real‐time motor and motor imagery decoding. a) Schematic of paradigms for intraoperative control of the µECoG BCI for real‐time motor decoding. b) Real‐time motor decoding performance of the intraoperative experiment. c) Reconstruction of functional brain areas based on fMRI under motor imagery paradigms. d) Approximate location of the µECoG electrode array on the right cortical surface of the participant.^[^ ^38^ ^]^ e) The number of accumulated trials during the 12‐day in vivo experiment. f) Schematic of paradigms for short‐term control of the µECoG BCI for real‐time motor imagery decoding. g) Following 6‐day adaptation training, the participant demonstrated full volitional control of the µECoG BCI to perform the Center‐out paradigm, with the bit rate improving progressively throughout the experiment. h) Movement trajectories for the Center‐out paradigm in a single block. i) The bit rates of the participant controlling the µECoG BCI to perform the Webgrids paradigm across days. j) After completing adaptation training for the Center‐out and Webgrids paradigms, the participant utilized the µECoG BCI to engage in video games.

Building on validated long‐term biosafety and efficacy of the developed µECoG BCI, complemented by decoder design insights from animal studies, we conducted clinical intraoperative and short‐term in vivo trials. For the localization of brain functional regions, the patient was awakened during surgery and played the Ping‐Pong and Snake games (**Figure** **4**a). In the first 7 min, the patient used a joystick to play the games while µECoG signals and game events were collected as training data for the decoder. Then, the connection between the joystick and the computer was disconnected, allowing the games to be controlled solely via µECoG signals. In the Ping‐Pong game, one‐dimensional movements were decoded to control the upward and downward movements of the racket, and a mean accuracy of 0.90 was achieved (Movie S2, Supporting Information). For the Snake game, two‐dimensional kinematic decoding simultaneously governed movement direction and speed, representing increased complexity relative to the Ping‐Pong game due to task constraints and potential data imbalance (Movie S3, Supporting Information). Mean decoding accuracies reached 0.73 in the X‐direction and 0.79 in the Y‐direction (Figure 4b).

To demonstrate the µECoG BCI's clinical utility in restoring autonomy for motor‐impaired patients via assistive device control, we implemented high‐precision cursor tasks. Pre‐surgical fMRI localized motor imagery‐related cortices, while post‐implantation computed tomography (CT) reconstructed µECoG electrode positions onto cortical surfaces (Figure 4c,d). During the 12‐day in vivo experiment, a total of 25,412 trials were accumulated for a total duration of 19.87 hours (Figure 4e). These trials consist of the Center‐out paradigm (8 targets) and the Webgrids paradigm (255 targets) (Figure 4f). From day 7, the participant was able to fully volitionally control the cursor via the µECoG BCI (without any additional assistance) to perform the Center‐out paradigm. The bit rate continuously increased as the experiment proceeded, and the highest bit rate was up to 1.13 (Figure 4g,h; Table S2, Supporting Information). For the more complex Webgrids paradigm initiated on day 8, daily bit rates converged with practice, reaching a maximum of 4.15 on day 9 following interface optimization (Figure 4i). However, the convergence values of the bit rates were closely related to the participant's state and did not exhibit the same growth trend as the Center‐out paradigm. This may owe to the disparity in the number of selectable targets between the two paradigms, making it difficult for the participant to fully adapt within a short period. Ultimately, the participant achieved BCI‐controlled operation of video games, intelligent wheelchairs, and smart home systems (Figure 4j; Movie S4, Supporting Information).

## 3 Conclusion

In summary, we developed a chronically stable, high‐density flexible µECoG BCI system via MEMS fabrication, enabling acquisition of high‐spatiotemporal‐resolution signals. This system achieves iEEG‐comparable decoding performance with reduced invasiveness, establishing clinical translatability for µECoG‐based BCIs. The revealed correlation between electrode density and decoding performance provides a critical reference for µECoG electrode array design. Furthermore, the scalable density and throughput of µECoG BCIs offer efficient hardware solutions for precise lesion localization, cortical mapping, and even future home‐use neuroprosthesis control. We expect µECoG BCIs to enable fundamental investigations into neural decoding mechanisms. Long‐term µECoG systems restoring multifunctional neurological capacities – including motor and communication functions – promise transformative clinical neurorehabilitation pathways.

## 4 Experimental Section

### Fabrication of the µECoG Electrode Arrays

The high‐density µECoG electrode arrays were fabricated using a sandwich structure with gold contacts embedded in flexible polyimide (PI) sheets, following a process similar to our previous work (Silk‐enabled conformal multifunctional bioelectronics for investigation of spatiotemporal epileptiform activities and multimodal neural encoding/decoding. 2019 AS).^[^ ^28^ ^]^ First, a 7 µm‐thick layer of PI (PI‐2610, HD Microsystems, USA) was spin‐coated onto a silicon wafer. After patterning a photoresist layer (AZ 5214), it used electron beam evaporation to deposit a metal layer consisting of 50 Å of chromium and 150 nm of gold. This was followed by a lift‐off procedure in acetone to define the recording sites and interconnectors. It then spin‐coated and cured an additional 13 µm‐thick PI layer to serve as the encapsulation layer. Next, aluminum was sputtered onto the surface as an etching mask and patterned using UV photolithography. This was followed by aluminum corrosion and oxygen plasma dry etching to create openings and perfusion holes. The fabrication process concluded with etching using hydrofluoric acid to release the device from the silicon wafer. The final electrode array features 16 × 16 recording sites, each with a diameter of 850 µm and spaced 1250 µm apart.

### Integration of the Implant

The integrated implant comprises four main components: a µECoG electrode array, a FPC, a signal processing unit, and a titanium enclosure. The core component was the µECoG electrode array, which provides conformal contact with the cortex, enabling acquisition of raw high‐resolution µECoG signals. The electrode array was bonded to the FPC to facilitate signal transmission and allow simultaneous addressing and acquisition of 256 electrode channels. The signal processing unit employs four Intan RHD2164 chips, which effectively suppress noise and amplify signals while maintaining low power consumption. Finally, a customized titanium enclosure serves as the encapsulation for the integrated implant, ensuring excellent waterproofing, outgassing resistance, and biocompatibility. This design guarantees the stable performance of the µECoG BCI during long‐term operation.

### Long‐Term In Vivo Experiment

To validate the long‐term safety and effectiveness of the µECoG BCI for real‐time motor decoding, it conducted the in vivo experiment in a canine model. A male Labrador dog, ≈18 months old and weighing 30 kg was selected. The Labrador dog was provided by Pharma Legacy Diagnostic Laboratories (Shanghai, China) Co., Ltd, a laboratory animal institution accredited by China National Accreditation Service for Conformity Assessment (CNAS LA0026), and the experimental protocol was approved by the Institutional Animal Care and Use Committee (IACUC) of this institution, with approval number PLJC22‐0184‐1. Following post‐operative recovery, the dog's motor functions and physiological parameters were assessed and determined to be within normal ranges. Kinematic features were obtained using two methods: first, by strapping an inertial measurement unit (IMU) above the dog's knee, which transmitted time‐stamped kinematic features to a computer via Bluetooth; second, by extracting kinematic features from multiple joints using video data, employing motion capture technology and DeepLabCut.^[^ ^29^ ^]^

### Immunohistochemistry Studies

At the conclusion of the observation period, the brain tissue samples of the dog were collected for in vitro analysis. Wet brain tissues were trimmed to ≈1.0 cm × 1.0 cm × 0.2 cm and placed into labeled embedding boxes. The tissues were then dehydrated using a tissue dehydrator, subjected to a gradient of alcohol, cleared with xylene, and finally embedded following gradient paraffin infiltration. Hematoxylin and eosin (H&E) staining was performed, utilizing 1% eosin dye. Additional tissue sections were incubated with specific antibodies: glial fibrillary acidic protein (GFAP) (targeting astrocytes, Abcam ab7260, USA), ionized calcium‐binding adapter molecule 1 (Iba‐1) (targeting microglia, Abcam ab107159, USA), and neuronal nuclei antigen (NeuN) (targeting neuronal nuclei, Abcam ab177487, USA). The immunohistochemically stained sections were photographed using a microscope to facilitate comparisons and visualize the effects of the implanted µECoG electrode array on brain tissue.

### Intraoperative Experiment

Participants in the study were involved only when there was a clinical necessity for awake surgery to safely resect a tumor and protect the M1 area. Prior to the procedure, each participant conformed that their involvement in the research was entirely voluntary and that the task was conducted for research purposes. Each participant received comprehensive information and provided written informed consent. The study received ethical approval from the Huashan Hospital Institutional Review Board of Fudan University (Approval No.: KY2021‐918) and was conducted according to the guidelines of all relevant ethical regulations.

It designed two intraoperative games: the Ping‐pong game and the Snake game. In the Ping‐pong game, the patient used rackets located on the left and right sides of the screen to catch a ping‐pong ball moving at a constant speed. The rackets could only be moved up or down. The game was considered failed if the ping‐pong ball touched the left or right edge of the screen. In the Snake game, various fruits appeared randomly on the screen, and the patient controlled a snake to move in four directions—up, down, left, and right—to collect them. The Snake game required decoding µECoG signals into kinematic features in two dimensions, and the game failed if the snake touched itself.

### Clinical Short‐Term In Vivo Experiment

Participants in the study were involved only when there was a clinical necessity for preoperative epilepsy monitoring. Prior to the procedure, each participant conformed that their involvement in the research was entirely voluntary and that the task was conducted for research purposes. Each participant received comprehensive information and provided written informed consent. The study received ethical approval from the Huashan Hospital Institutional Review Board of Fudan University (Approval No.: KY2024‐603) and was conducted according to the guidelines of all relevant ethical regulations.

It designed the Center‐out paradigm and the Webgrids paradigm. In these paradigms, the participant should move the cursor to the target highlighted on the screen within 4 seconds and hold on for 200 ms; otherwise, the trial failed. In order to enable the participant to quickly adapt to controlling the cursor through motor imagery, a progressive training method was used.^[^ ^30^ ^]^ During the first six days of the experiment, a flexible fixturing algorithm was used to enhance the participant's experience of control.^[^ ^31^ ^]^ From the day 7, the participant was able to control the cursor leveraging motor imagery without any additional assistance after calibration ≈30 min.

### Neural Recording

The high spatiotemporal resolution µECoG signals from our µECoG electrode arrays were amplified and digitized by a signal processing unit connected to a 1024‐channel data acquisition system, the CereCube NSP8 (Neuroxess Co., Ltd.). In the long‐term in vivo experiment, the reference and ground electrodes were placed on the dura mater and skull of the dog, and similarly, in the intraoperative experiment, the reference and ground electrodes were positioned on the dura mater and skull of the patient as well. µECoG signals were recorded at a sampling rate of 4000 Hz during all sessions. The files containing µECoG signals were timestamped with start and end times to facilitate alignment with other data. To calculate the SNR, the high and low activity states of the ECoG were distinguished and the ratio of the peak‐to‐peak value of the high activity state to the RMS value of the low activity state was calculated.^[^ ^32^ ^]^

### Feature Extraction and Motor Decoder

Before processing the µECoG signals, channels with impedance greater than 1 MΩ were excluded based on pre‐session measurements.^[^ ^33^ ^]^ Notch filters were applied to attenuate the power frequency of 50 Hz and its harmonics. A common average reference was used across all electrode channels to reduce artifacts and common mode noise. The preprocessed µECoG signals were bandpass filtered to prevent frequency aliasing and down sampled to 500 Hz to reduce the time required for subsequent analysis. The µECoG signals were then binned into 100 ms segments, and PSD was calculated (Figures S22 and S23, Supporting Information). Mean values of the HG band PSDs were extracted as features indicative of movement‐related neural activity (Figures S24 and S25, Supporting Information).^[^ ^34^ ^]^ Before feeding the training data into the decoder, the PSD features and real kinematic features (or game events) were Z‐score normalized and aligned based on timestamps.

It employed a LSTM recurrent neural network (RNN) with three cascaded LSTM layers for motor decoding. The input to the LSTM decoder was a row vector containing the PSD features from the electrode channels at each time step. The output sizes for each LSTM layer were 300, 100, and 50, respectively. A fully connected layer served as the output layer, mapping the last hidden states to the kinematic features. For training the LSTM decoder, it applied L2 normalization as the penalty function, with an initial learning rate of 1e‐3. The dataset was divided into training and test sets in a 7:3 ratio. To mitigate overfitting, it employed weight decay and early stopping strategies. The LSTM decoder can still achieve high‐accuracy decoding even with a large number of channel loss, demonstrating its robustness (Figure S26, Supporting Information). In the short‐term in vivo experiment, a position‐velocity Kalman filter was designed to be used for motor imagery decoding (Figure S27, Supporting Information).^[^ ^35^ ^]^

### Performance Indicators of Motor and Motor Imagery Decoding

For the motor decoding, each sample of the decoder output represents the kinematic features encoded in the ECoG within 100 ms bins. To quantify the accuracy of motor decoding, it calculated Pearson's correlation coefficients between predicted and actual trajectories. To gain a comprehensive view of decoding accuracy, correlation coefficients were assessed at different time scales. For evaluating the decoding performance of various electrode subsets, correlation coefficients were computed across the entire session. A sliding time window was utilized, resulting in an in‐session accuracy curve to analyze the short‐term stability of motor decoding.

For the motor imagery decoding, the bit rate (BR) was calculated after each trial, according to the formula as follows:^[^ ^18^ ^]^

(1) $$BR=log_{2}N\cdot \frac{max\left(S-F,0\right)}{T}$$

where *N*, *S*, *F*, *T* are the number of selectable targets, successful trials, failed trials, and the total time for performing the paradigms, respectively. Trials exceeding the time limit were considered failed, with the time consumed included in the denominator.

### Contribution of µECoG Electrodes to Motor Decoding

The contribution of µECoG electrodes to motor decoding was assessed to explore the spatial structure of ECoG‐encoded kinematic features. It employed a two‐stage algorithm to evaluate the contribution of each electrode channel. In the first stage, a random forest model was constructed using HG features and labeled kinematic features. The feature importance for each electrode channel was calculated based on the normalized reduction of Gini impurity. In the second stage, it created a subset of electrode channels starting with the five channels that had the highest importance. This subset was used for motor decoding, and additional electrode channels were added. This process allowed to generate an accuracy curve for motor decoding as the number of electrode channels in the subset increased. The channels corresponding to the peak of the accuracy curve were considered to have contributed to motor decoding in each session.

### Statistical Analysis

To assess signal sharing between µECoG electrodes, it calculated the cross‐electrode cross‐correlation function.^[^ ^36^ ^]^ The processed signals were divided into 2‐second bins, and the peak values of the cross‐correlation functions were recorded, creating similarity curves for each electrode channel. Z‐score normalization was applied to each electrode channel to eliminate the effects of amplitude differences. Before performing the significance tests, it assessed the samples for normality and variance. The paired t‐test was applied to two groups of samples that followed a normal distribution with equal variances; otherwise, the Wilcoxon rank‐sum test was utilized. All data analyses were performed using MATLAB (R2024a, MathWorks).

## Conflict of Interest

The authors declare no conflict of interest.

## Author Contributions

E.Z., and X.W. contributed equally to this work. Z.Z., T.H.T., Z.W., and L.C. conceived the idea. Z.Z., T.H.T., L.C., E.Z., L.P., and Y. M. designed the experiments. X.W. and Z.Z. fabricated the µECoG electrode arrays. Z.Z., J.L., X.W., and E.Z. design the integrated implantation. Z.Z., E.Z., X.W., Y.L., J.L., Q.Y., X.R., L.X., Z.W., X.Z., and L.C. performed the experiments. E.Z., Z.Z., Q.Y., X.R., L.X., C.L., L.S. analyzed the data. E.Z., Z.Z., and T.H.T. wrote the paper. All authors discussed the results and provided comments for the manuscript.

## Supporting information



Supporting Information

Supplemental Movie 1

Supplemental Movie 2

Supplemental Movie 3

Supplemental Movie 4

## Data Availability

The data that support the findings of this study are available on request from the corresponding author. The data are not publicly available due to privacy or ethical restrictions.
