# Supplementary material for: Chronically Stable, High‐Resolution Micro‐Electrocorticographic Brain‐Computer Interfaces for Real‐Time Motor Decoding
Source: Adv Sci (Weinh). 2025 Sep 6;12(45):e06663. doi: 10.1002/advs.202506663 (PMC12677598; doi:10.1002/advs.202506663)
Supplement: Supplementary file 1 — Supporting Information [file ADVS-12-e06663-s004.docx]

Supporting Information

Chronically stable, high-resolution micro-electrocorticographic brain-computer interfaces for real-time motor decoding

Erda Zhou^†^, Xiner Wang^†^, Jizhi Liang, Yang Liu, Qinrong Yang, Xingchen Ran, Lei Xia, Xiang Zou, Changjiang Liu, Liuyang Sun, Lei Peng, Liang Chen, Ying Mao, Zehan Wu*, Tiger H. Tao* and Zhitao Zhou*

Erda Zhou, Xiner Wang, Jizhi Liang, Changjiang Liu, Prof. Liuyang Sun

2020 X-Lab, Shanghai Institute of Microsystem and Information Technology, Chinese Academy of Sciences, Shanghai 200050, China

Prof. Zhitao Zhou

State Key Laboratory of Transducer Technology, Shanghai Institute of Microsystem and Information Technology, Chinese Academy of Sciences, Shanghai 200050, China
E-mail: [ztzhou@mail.sim.ac.cn](mailto:ztzhou@mail.sim.ac.cn)

Erda Zhou, Xiner Wang, Jizhi Liang, Prof. Zhitao Zhou

School of Graduate Study, University of Chinese Academy of Sciences, Beijing 100049, China

Yang Liu, Qinrong Yang, Xingchen Ran, Lei Xia, Lei Peng, Prof. Tiger H. Tao

Neuroxess Co., Ltd., Shanghai 200023, China

E-mail: tiger@mail.sim.ac.cn

Xiang Zou, Prof. Liang Chen, Prof. Ying Mao, Zehan Wu

Department of Neurosurgery, Huashan Hospital of Fudan University; Shanghai 200040, China

E-mail: zhwu08@fudan.edu.cn

Prof. Liuyang Sun, Prof. Zhitao Zhou

School of Integrated Circuits, University of Chinese Academy of Sciences, Beijing 100049, China

Prof. Tiger H. Tao

Guangdong Institute of Intelligence Science and Technology, Hengqin, Zhuhai, Guangdong 519031, China

Prof. Tiger H. Tao

Tianqiao and Chrissy Chen Institute for Translational Research, Shanghai, China.

**Keywords:** Brain-computer interfaces, High-resolution micro-electrocorticography, Real-time motor decoding, Flexible conformal micro-electro-mechanical systems


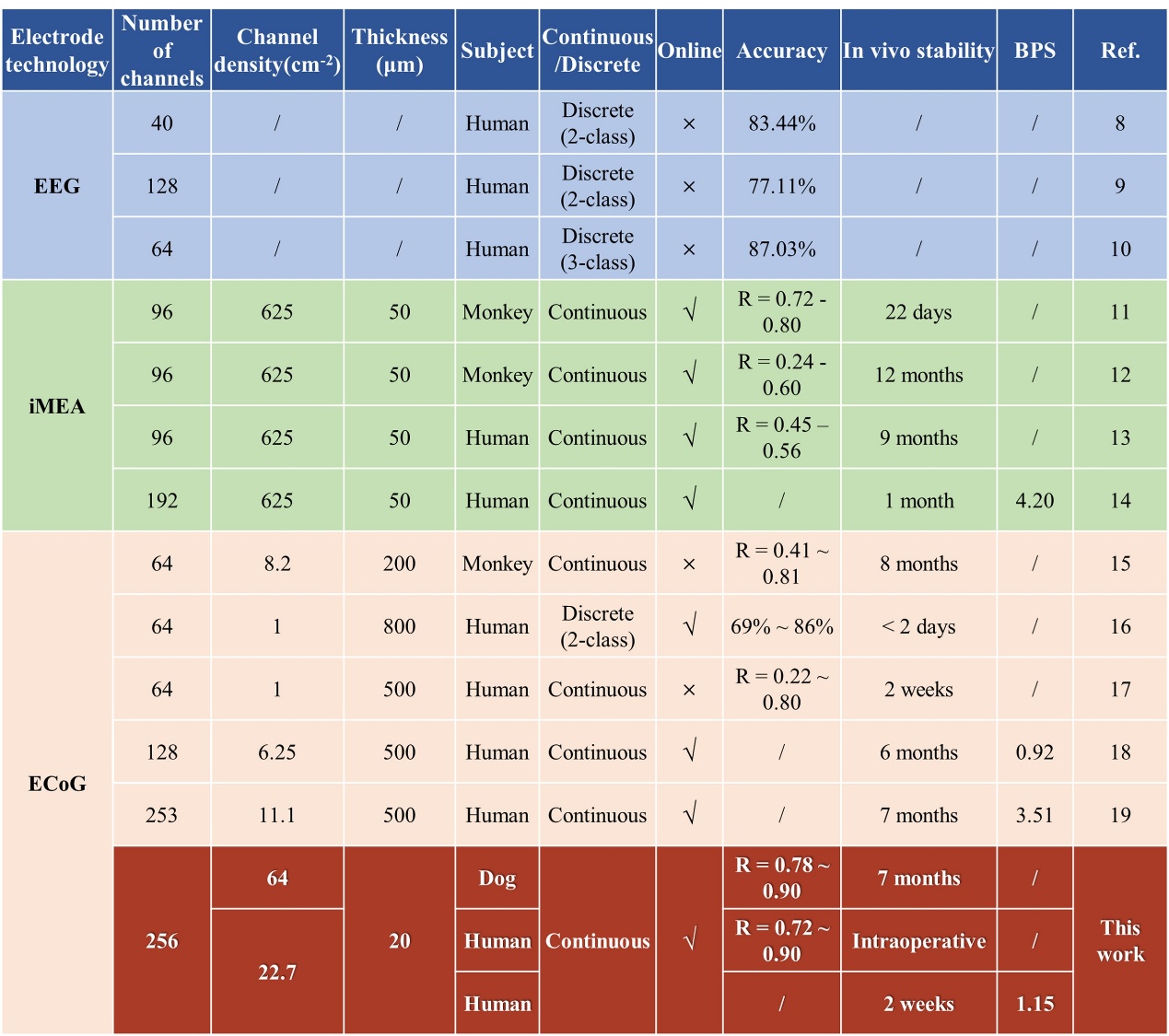


**Table S1.** Review of current and our μECoG BCIs. Review of studies using EEG, iEEG and ECoG acquired by EEG electrodes, iMEA and ECoG electrode arrays for motor and motor imagery decoding. IEEG has ultra-high spatio-temporal resolution with high accuracy and stability. However, iMEA involves intracortical implantation that causes damage to the brain, which is highly invasive. In contrast, non-invasive EEG is temporarily unable to achieve satisfactory decoding performance and continuous real-time decoding is difficult. ECoG, which is a compromise between EEG and iEEG, is capable of continuous real-time decoding, and the performance is better than EEG but slightly inferior to iEEG. We achieved a decoding performance comparable to that of iEEG using ECoG by increasing electrode density with conformal adherence capability.

**
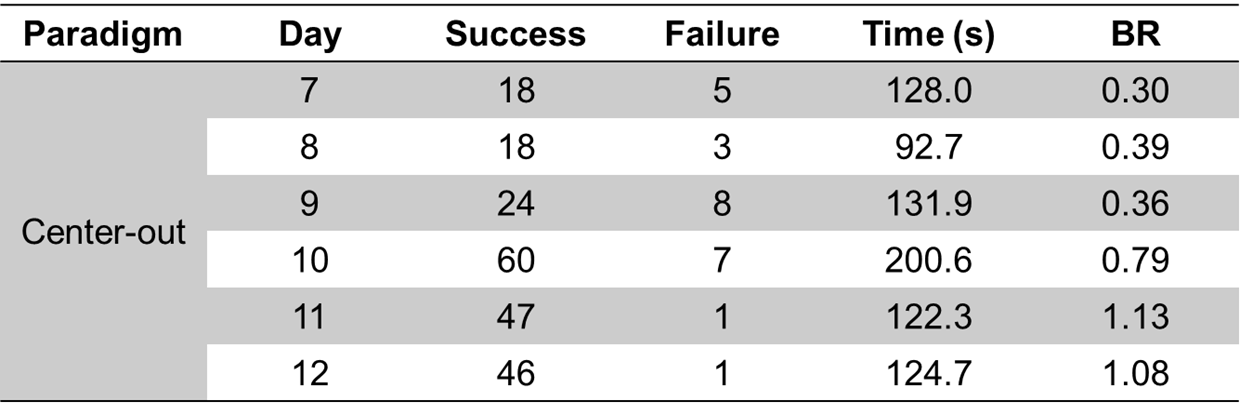
**

**Table S2.** The detailed data used for BR calculation in the Center-out Paradigm.


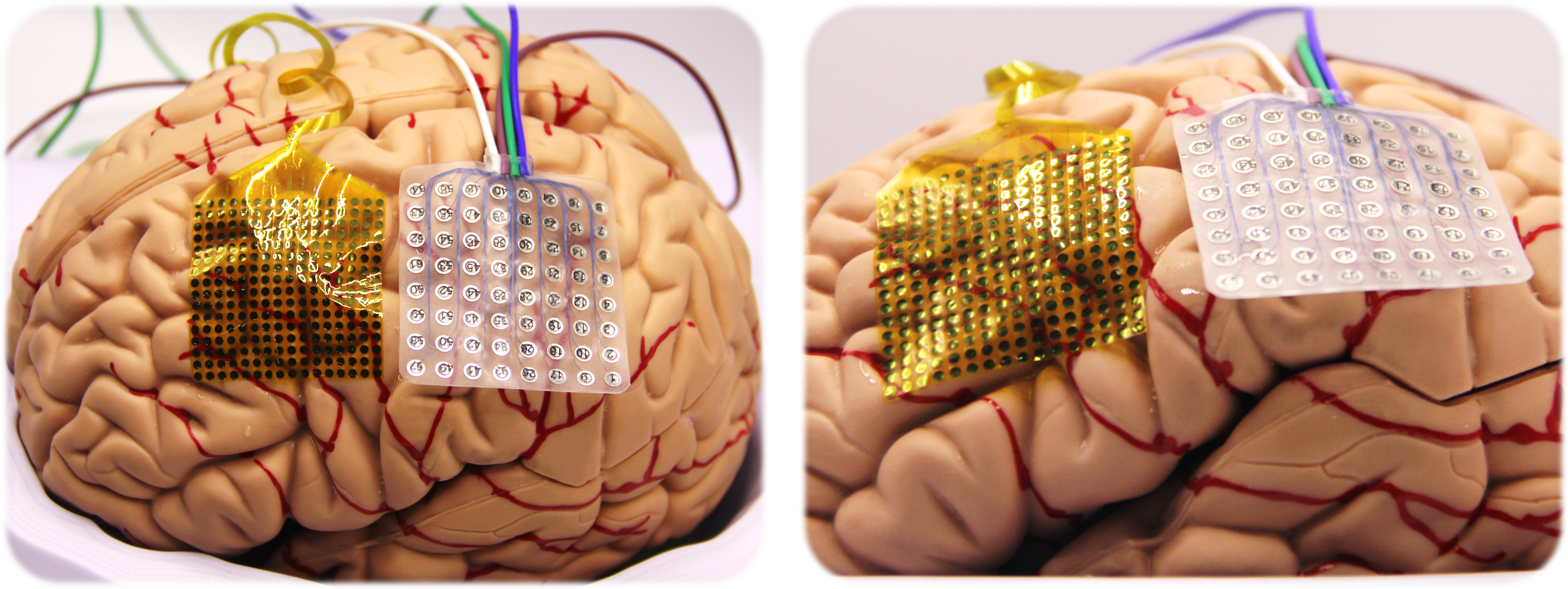


**Figure S1.** The adhesion performance of conventional clinical ECoG and our μECoG electrode arrays.


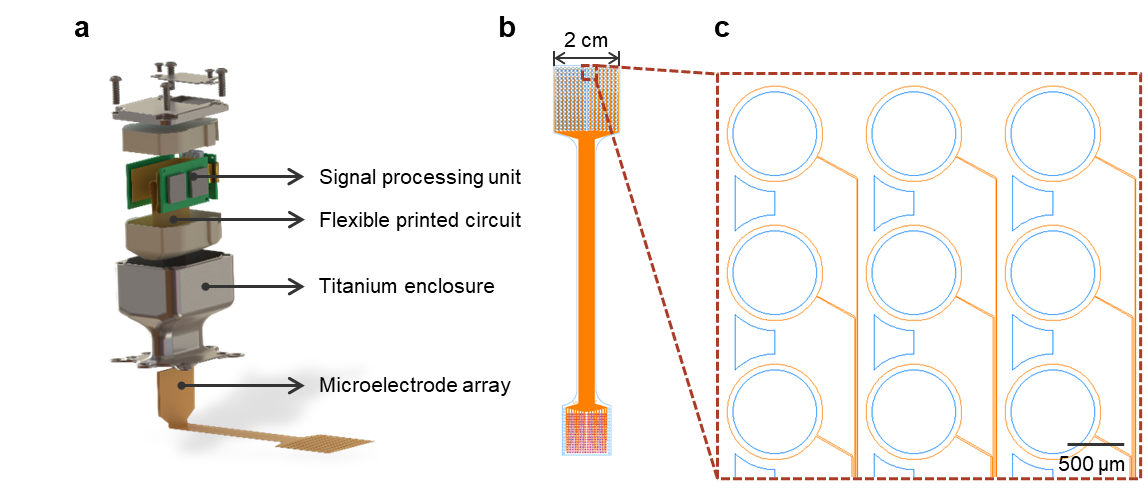


**Figure S2.** Design of implants and μECoG electrode arrays. a) Exploded view of the integrated implants. b) Overall layout of the 256-channel μECoG electrode arrays. c) Enlarged diagram depicting nine recording sites surrounded by mesh structures.


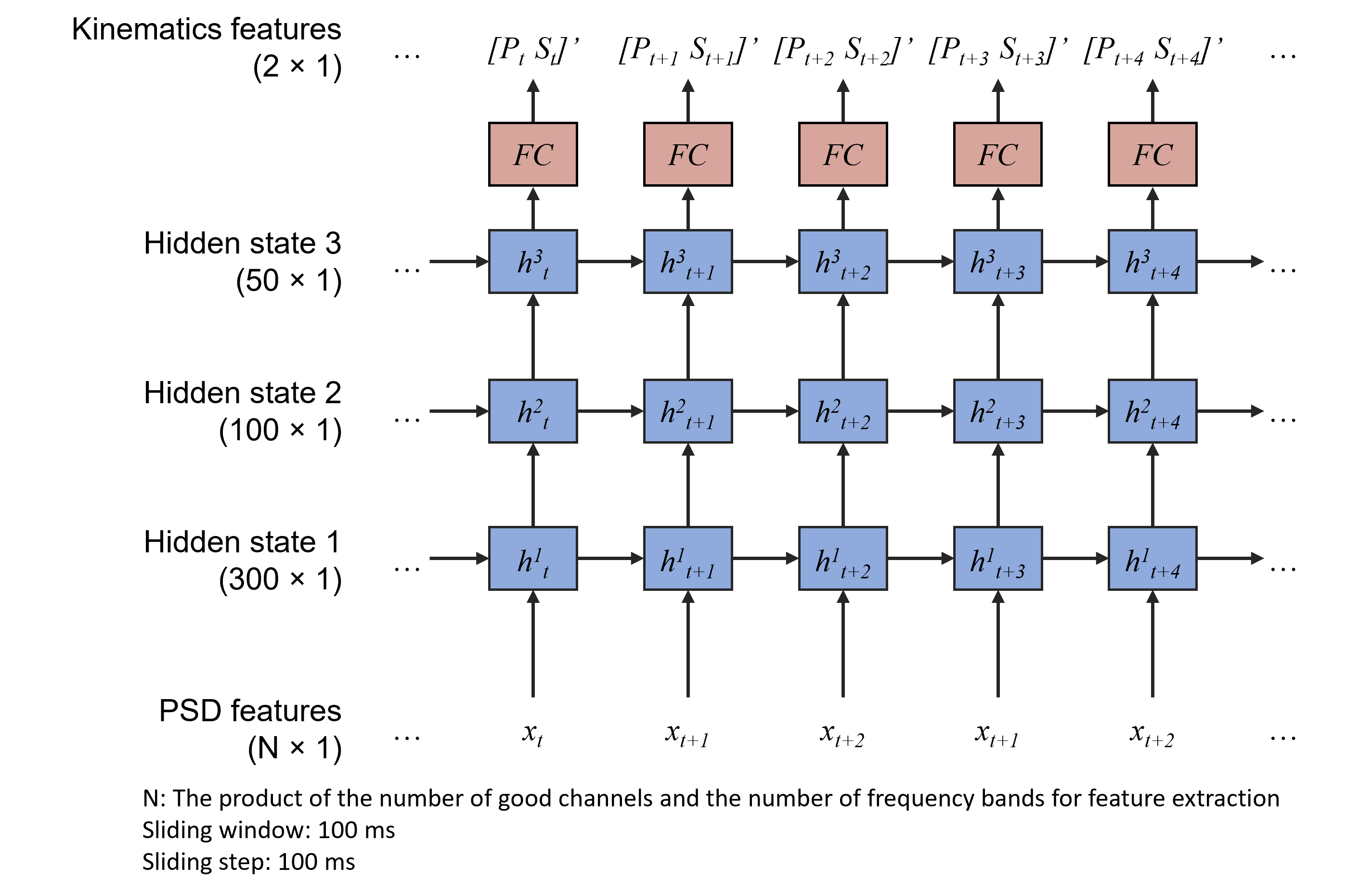


**Figure S3.** Network structure diagram of LSTM decoder. The kinematics features decoder utilizes a three-layer LSTM network. The sizes of the hidden states are 300, 100, 50. At the culmination of the process, a fully connected layer is applied to convert the third hidden state into kinematics features.

**
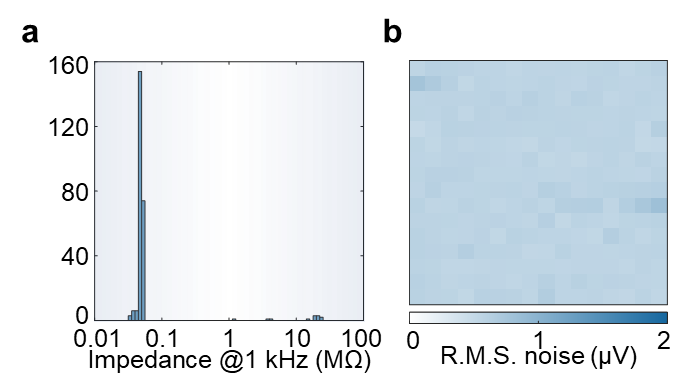
**

**Figure S4.** Characterization of the μECoG electrode array before implantation and the yield during long-term *in vivo* experiments. a) Histogram of electrode impedance at 1 kHz frequency for the μECoG electrode array before implant. b) Spatial distribution of RMS noise in phosphate buffer saline.

**
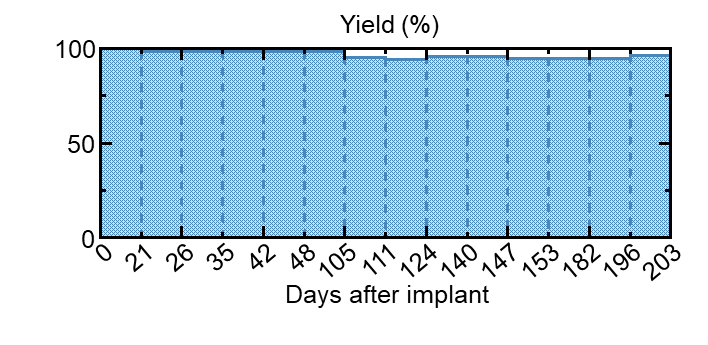
**

**Figure S5.** The yield of the μECoG electrode in *in vivo* experiments. During 203-day long-term *in vivo* experiments, the decrease in yield for the electrode channels was less than 10%, which indicates the reliability of the μECoG electrode arrays. The data were normalized based on the yield post-implantation.


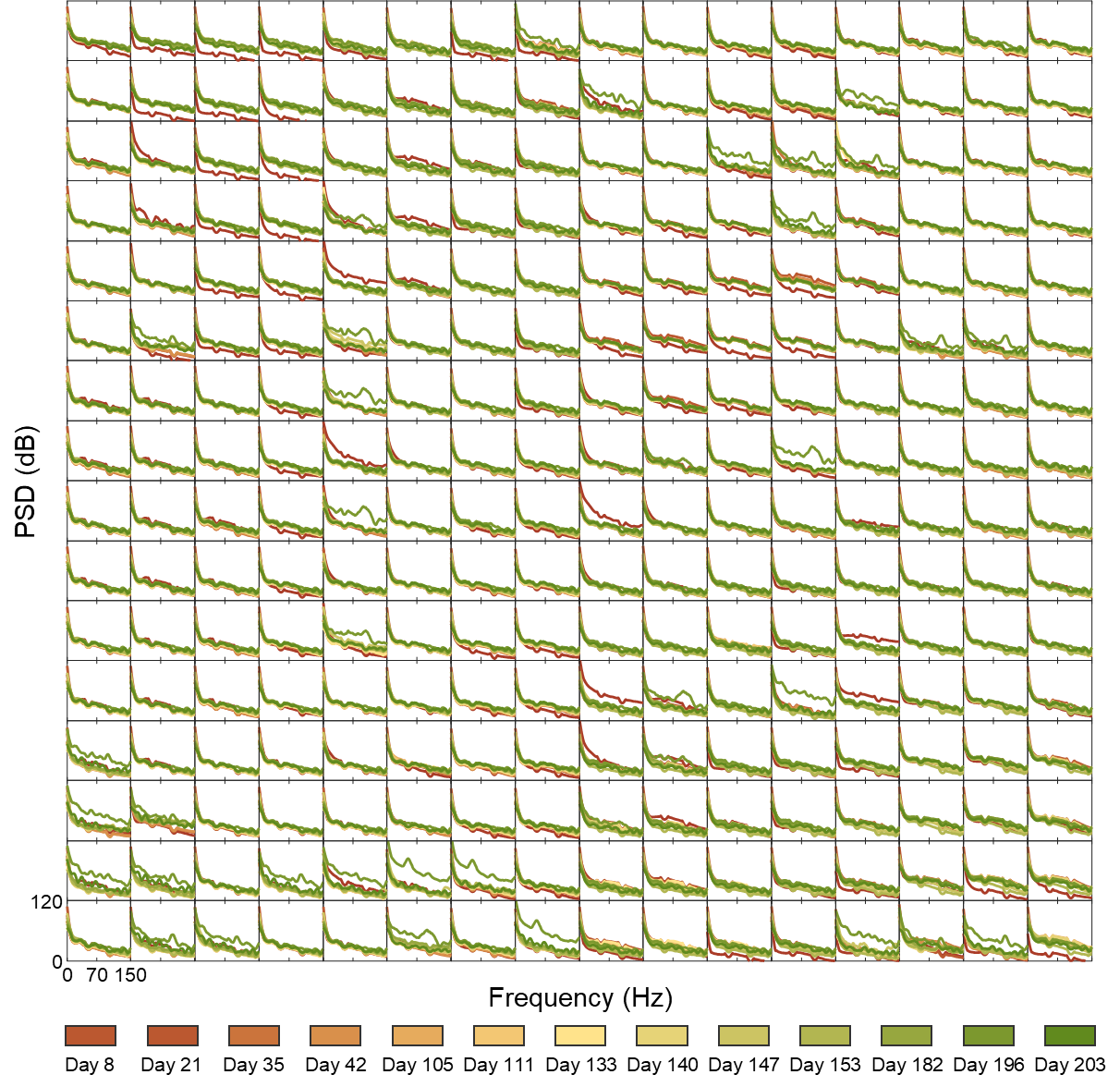


**Figure S6.** Spatio-temporal distribution of PSDs. In long-term in vivo experiments, the spatial distribution of the PSDs of the ECoG recordings acquired by each electrode channel was stable, indicating that the μECoG electrode arrays were not displaced relative to the cortex.


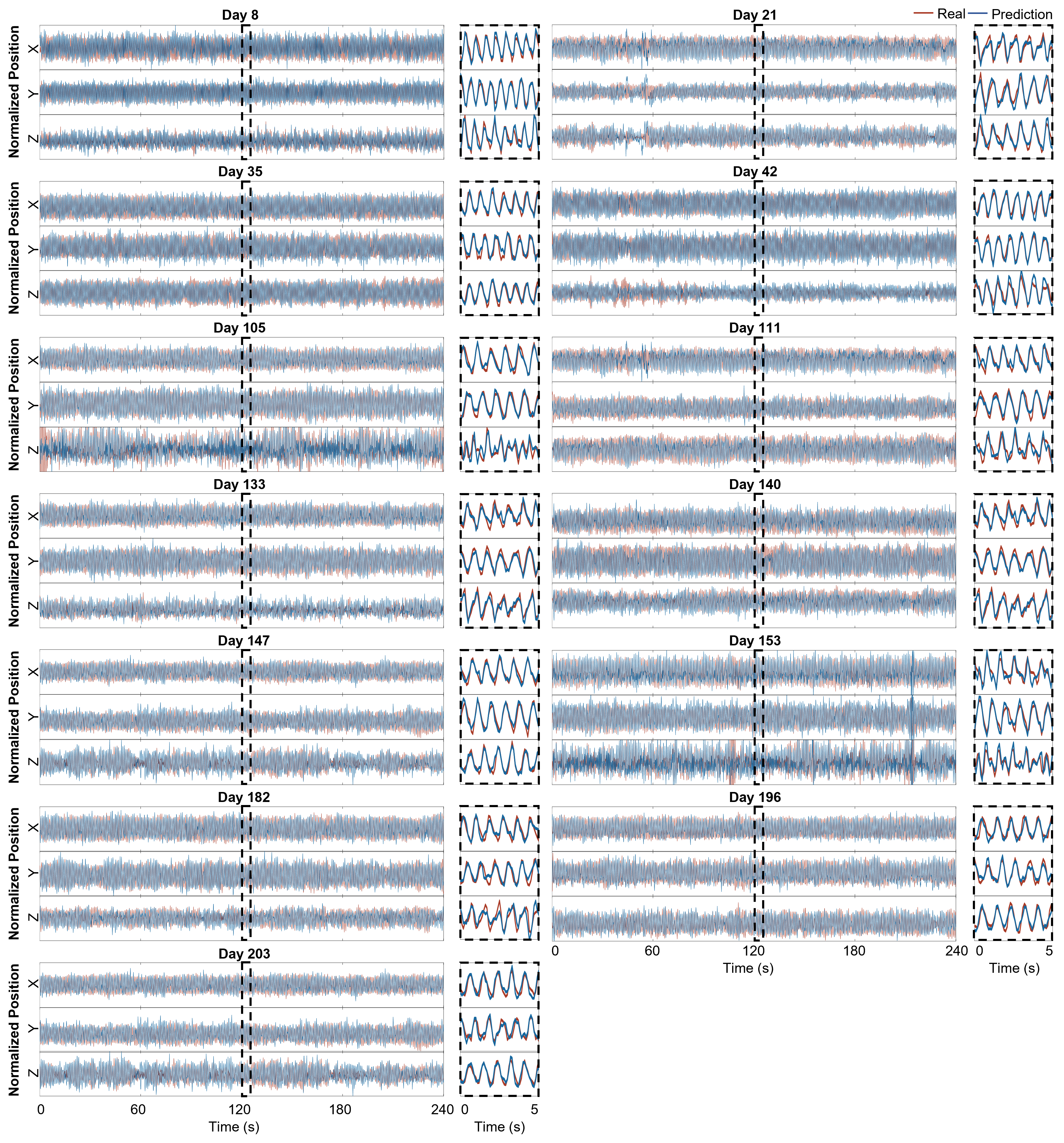


**Figure S7.** Predicted and real trajectories of position. Real-time position prediction results for 4 minutes within each session are displayed. Our μECoG BCI is capable of real-time tracking trends in real position, accurately predicting the trajectory with a low frequency of outliers.


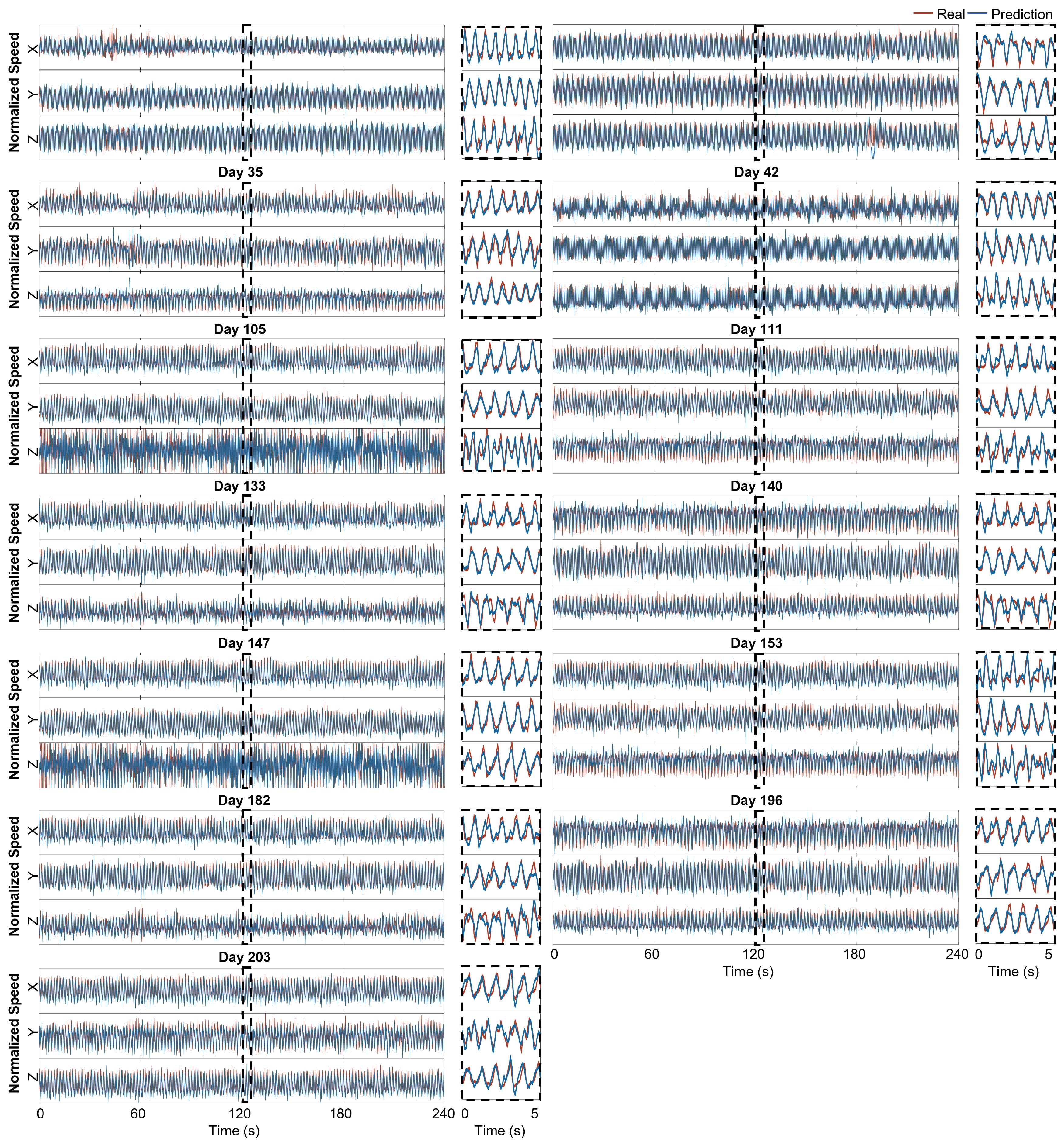


**Figure S8.** Predicted and real trajectories of speed. Real-time speed prediction results for 4 minutes within each session are displayed. Our μECoG BCI is capable of real-time tracking trends in real speed, accurately predicting the trajectory with a low frequency of outliers.


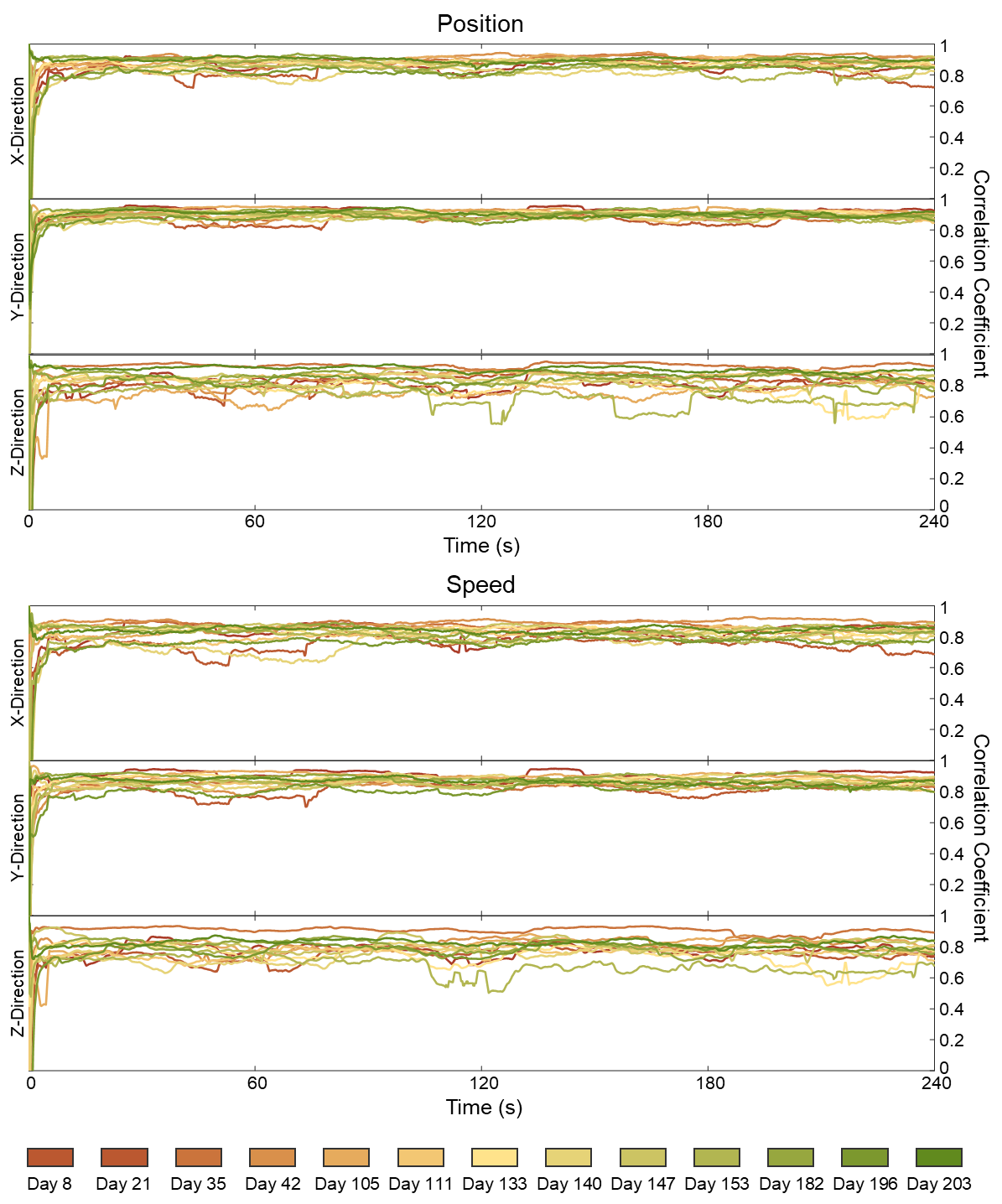


**Figure S9.** Correlation coefficient curves for all sessions. Decoding performance of the μECoG BCI is well stabilized on both short (within-session) and long (across-session) scales.

**
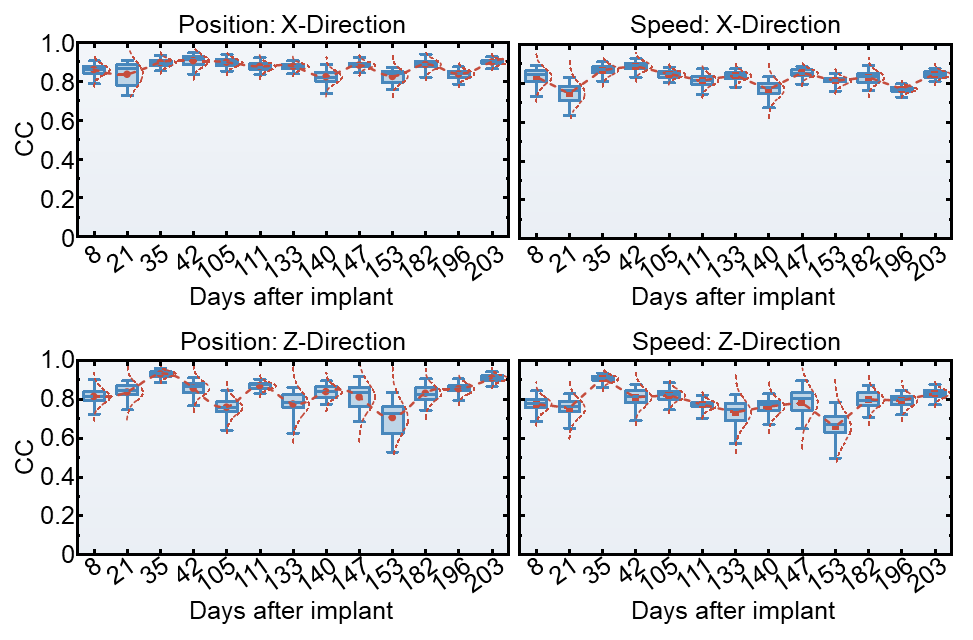
**

**Figure S10.** Long-term performance of motor decoding in the X and Z directions. In knee flexion and extension movements, movements in the X direction, which contribute to gait fluidity, have a smaller amplitude compared to those in the Y direction. Movements in the Z direction, which aid in balancing the body, have the smallest amplitude. The decoding accuracy in the X and Z directions, as demonstrated in in vivo experiments, showed consistency with these phenomena and exhibited long-term stability.


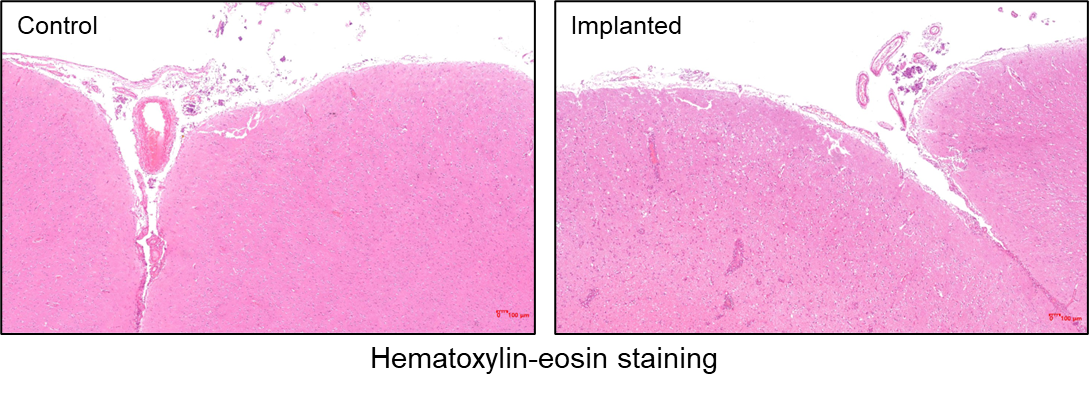


**Figure S11.** Optical images of hematoxylin-eosin staining of implantation sites and control sites. The nucleus was colored blue-purple using hematoxylin stain and the cytoplasm and extracellular matrix were colored pink using eosin stain. There was no significant difference in cell morphology between the area where the μECoG electrode array was implanted and the control area.


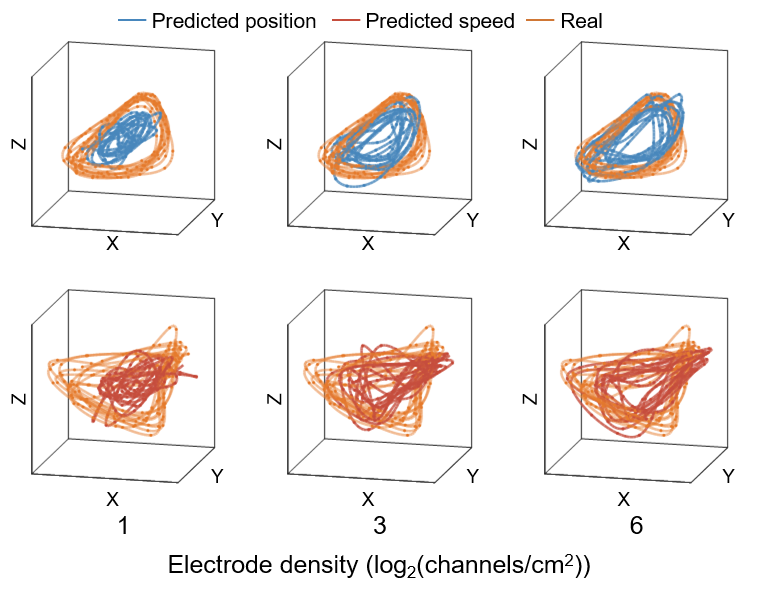


**Figure S12.** Changes in predicted trajectories of motion features as electrode density increases. Supplementing the predicted and real trajectories corresponding to the electrode density versus decoding performance curves in **Figure 2e**. Electrode density enhancement elevates the scale of variation of the predicted trajectories, attenuates the entangled state in 3-D space, and provides greater consistency with the real trajectories.


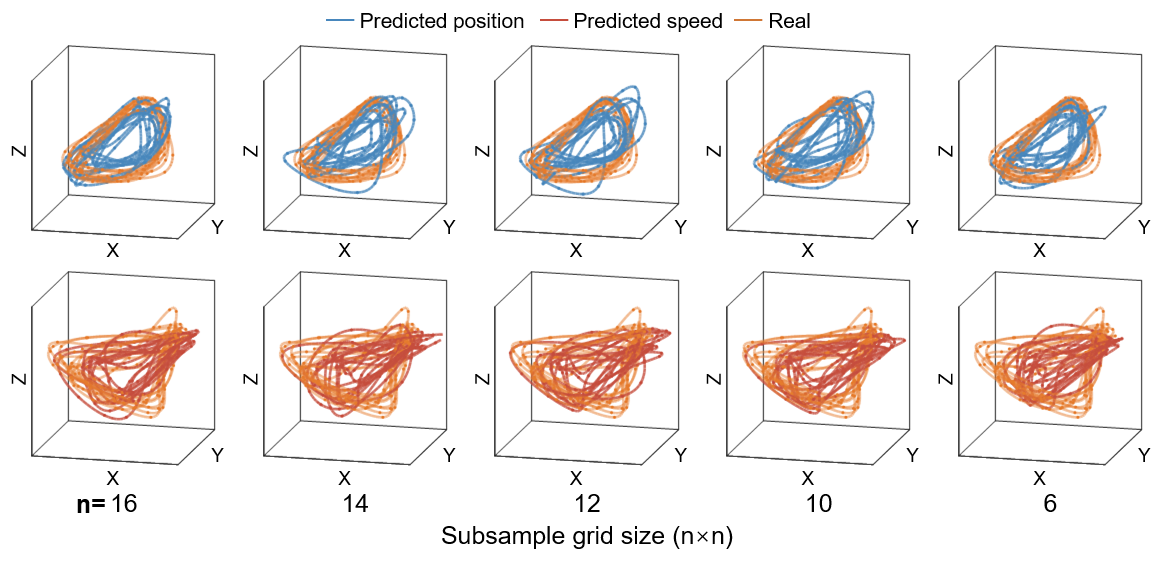


**Figure S13.** Changes in predicted trajectories of motion features as throughput increases. Supplementing the predicted and real trajectories corresponding to the subsample grid size versus decoding performance curves in **Figure 2f**. The expansion of brain coverage and electrode throughput show a significant boundary effect on the improvement of the consistency between predicted and real trajectories.


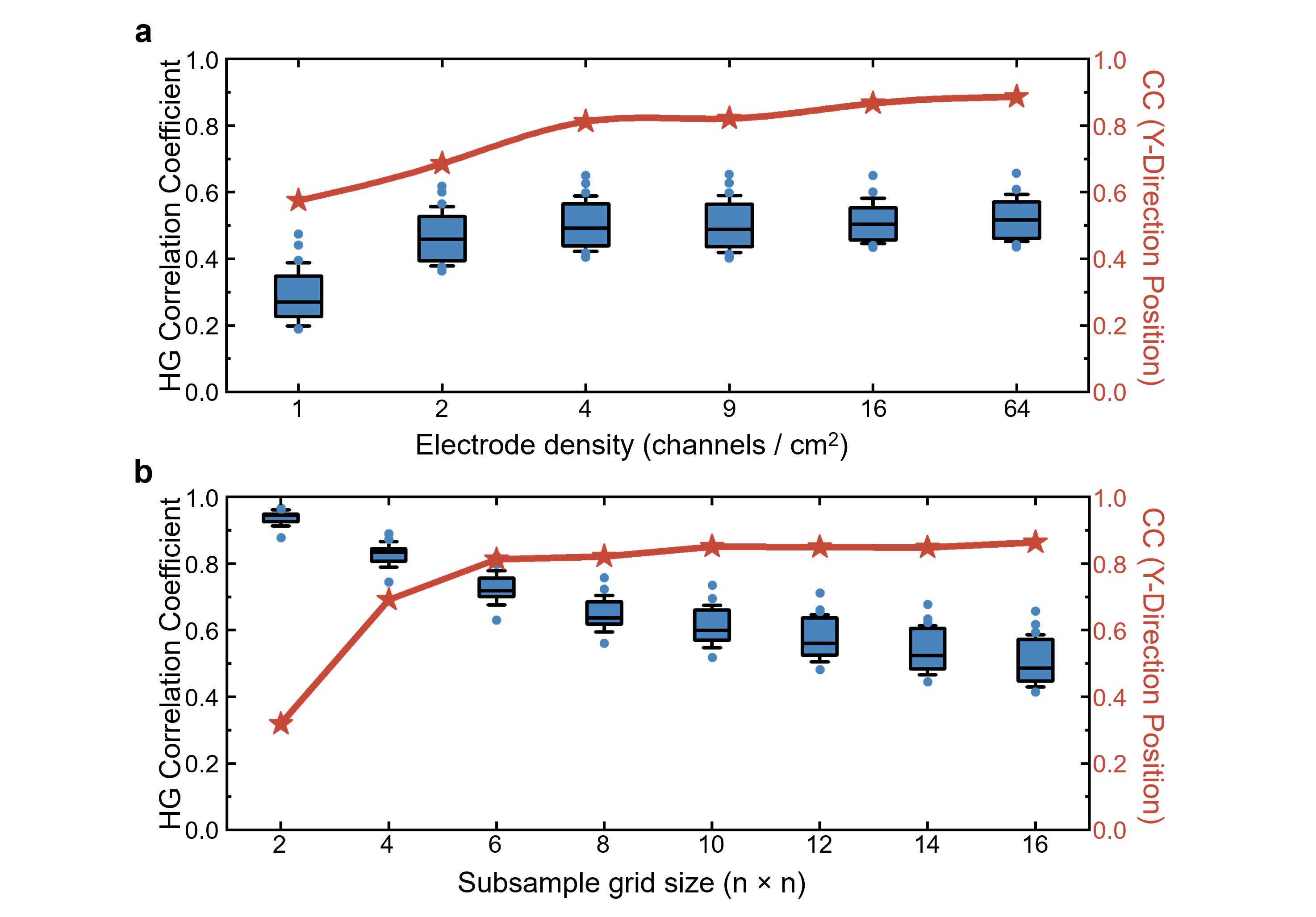


**Figure S14.** Relationship of HG band μECoG signals correlation coefficients and decoding accuracy to electrode density (top panel). Relationship of HG band μECoG signals correlation coefficients and decoding accuracy to subsample grid size (bottom panel). In the box plot, the boxes represent the IQR, with an internal line marking the median. The whiskers extend to cover the range of mean ± standard deviation.


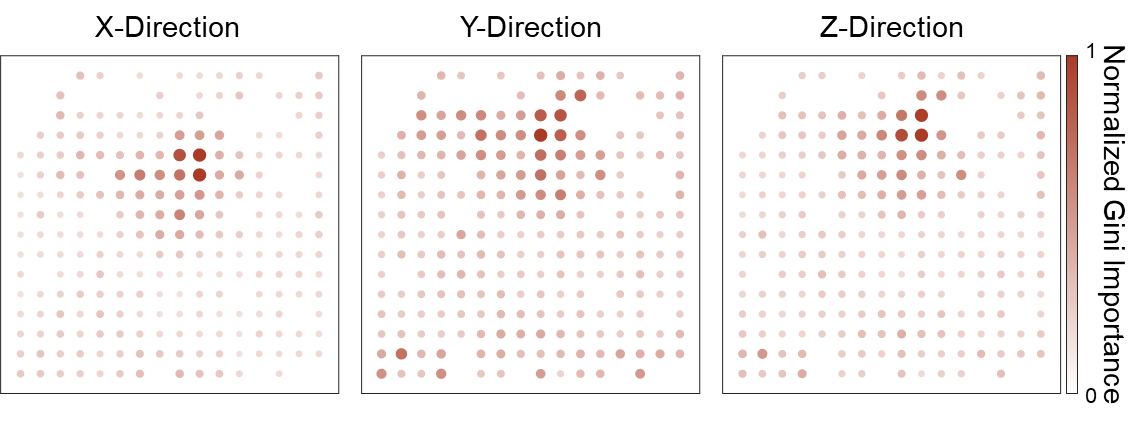


**Figure S15.** Spatial distribution of electrode Gini importance to motor decoding. There are differences in the spatial distribution of Gini importance for motor decoding in different directions. The high spatial resolution of the μECoG recordings enables precise searching for effective electrode channels and superior adaptability to different decoding scenarios.


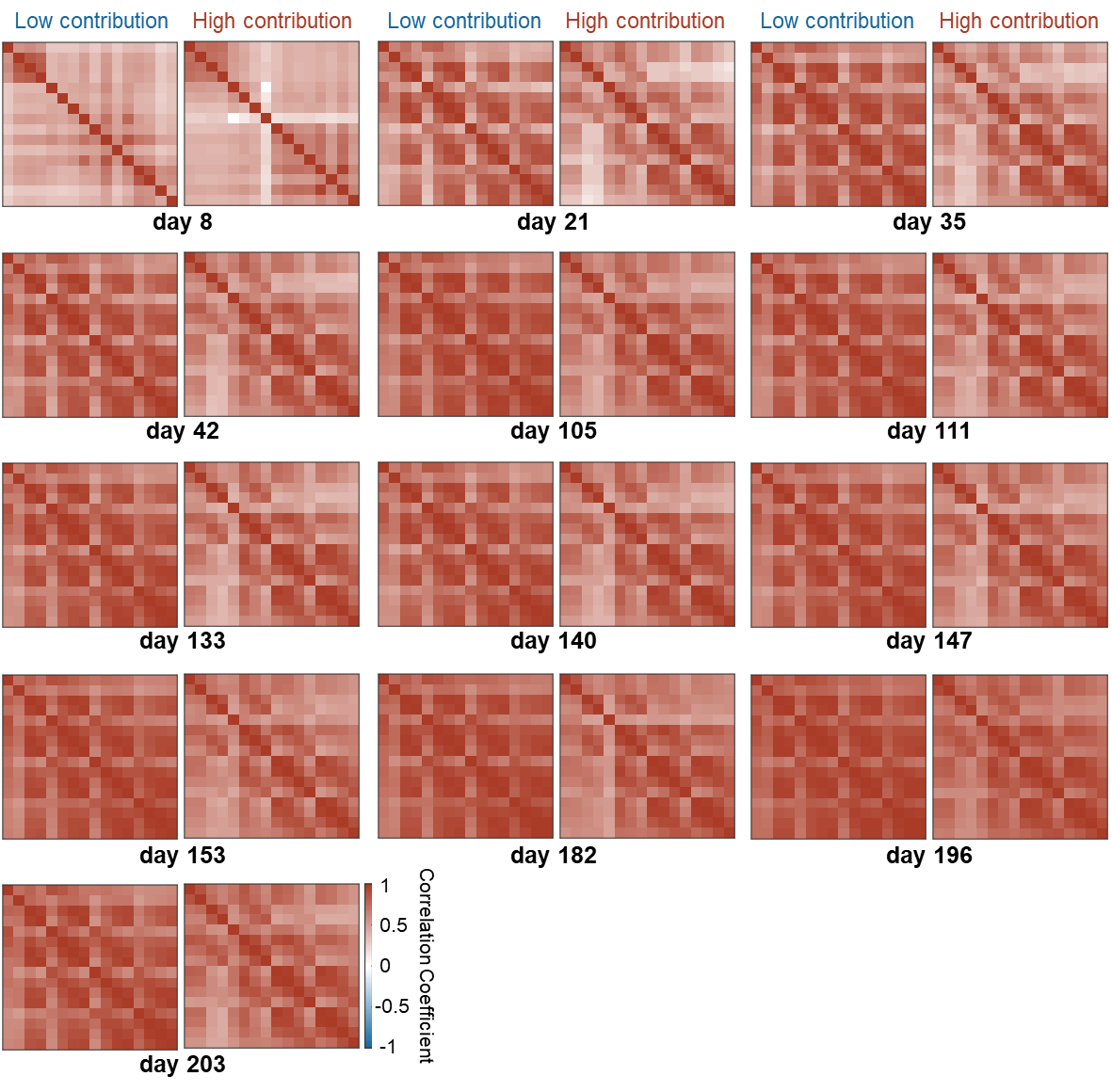


**Figure S16.** Inter-electrode correlation coefficients of HG band ECoG recordings for low and high contribution subsets. Inter-electrode correlation coefficient is a common measure of the similarity degree of μECoG signals, and the results show that the inter-electrode correlation is stronger for the subset of low contribution electrodes and vice versa for the subset of high contribution electrodes. This means that the high contribution subset may acquire more differentiated motor encoding information, which is beneficial for decoding. The correlation analysis of inter-electrode shows good agreement with the calculation of electrode contributions and the quantification of inter-electrode signal similarity.


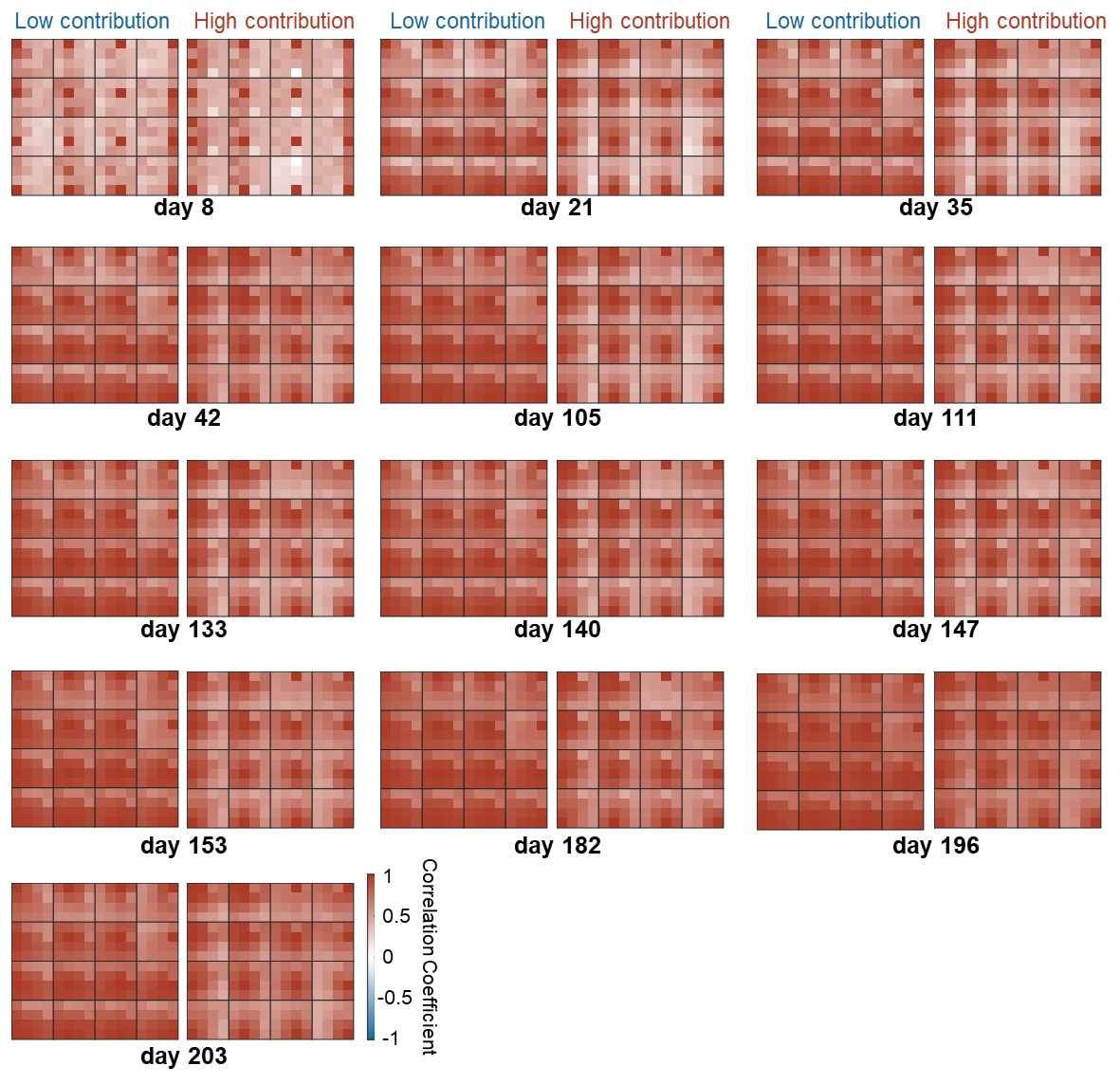


**Figure S17.** Spatial distribution of HG band inter-electrode correlation coefficients for low and high contribution subsets. Each block represents the correlation coefficients of HG band μECoG recordings acquired by electrode channels from low and high contribution subsets according to real relative spatial position. High contribution subset shows more pronounced spatial differences.


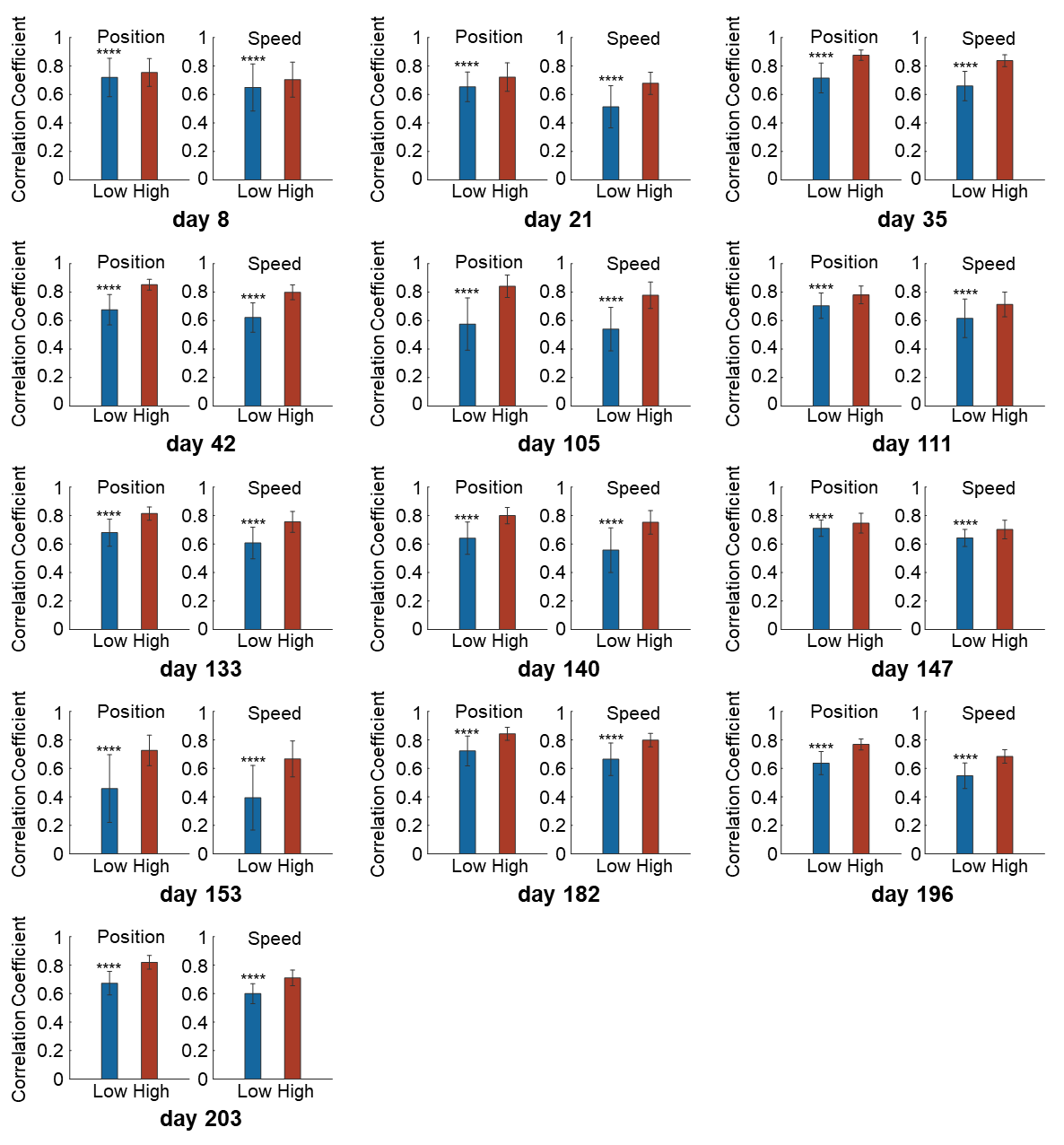


**Figure S18.** Performance of motor decoding using low contribution and high contribution subsets during 203 days. The bar plots show the decoding accuracy (means ± standard deviation) of the subset of low contribution versus high contribution electrodes (*p < 0.05, **p < 0.01, ***p < 0.001, ****p < 0.0001, n = 31200) within-sessions. For sufficient sample size, all sample points from all sessions are used here. In long-term in vivo motor decoding sessions, the subset of high contribution electrodes shows significantly better decoding ability than the low contribution subset.


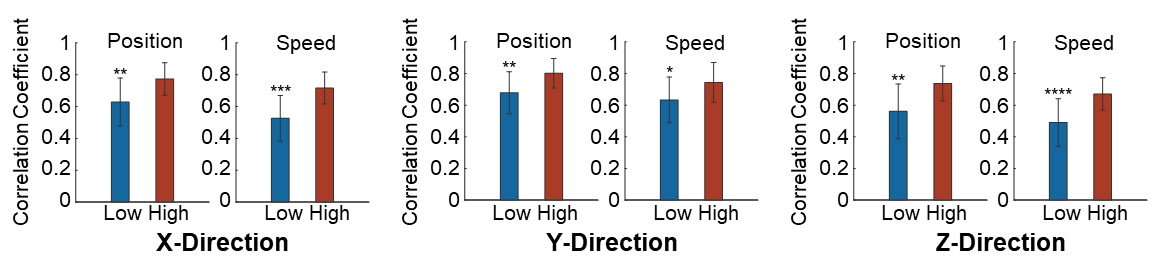


**Figure S19.** Motor decoding performance using low contribution and high contribution subsets in different directions. The bar plots show the decoding accuracy (means ± standard deviation) of the subset of low contribution versus high contribution electrodes in different directions (*p < 0.05, **p < 0.01, ***p < 0.001, ****p < 0.0001, n = 13) across sessions. The subset of high-contribution electrodes has a significant advantage in motor decoding in X, Y, and Z, all three directions.


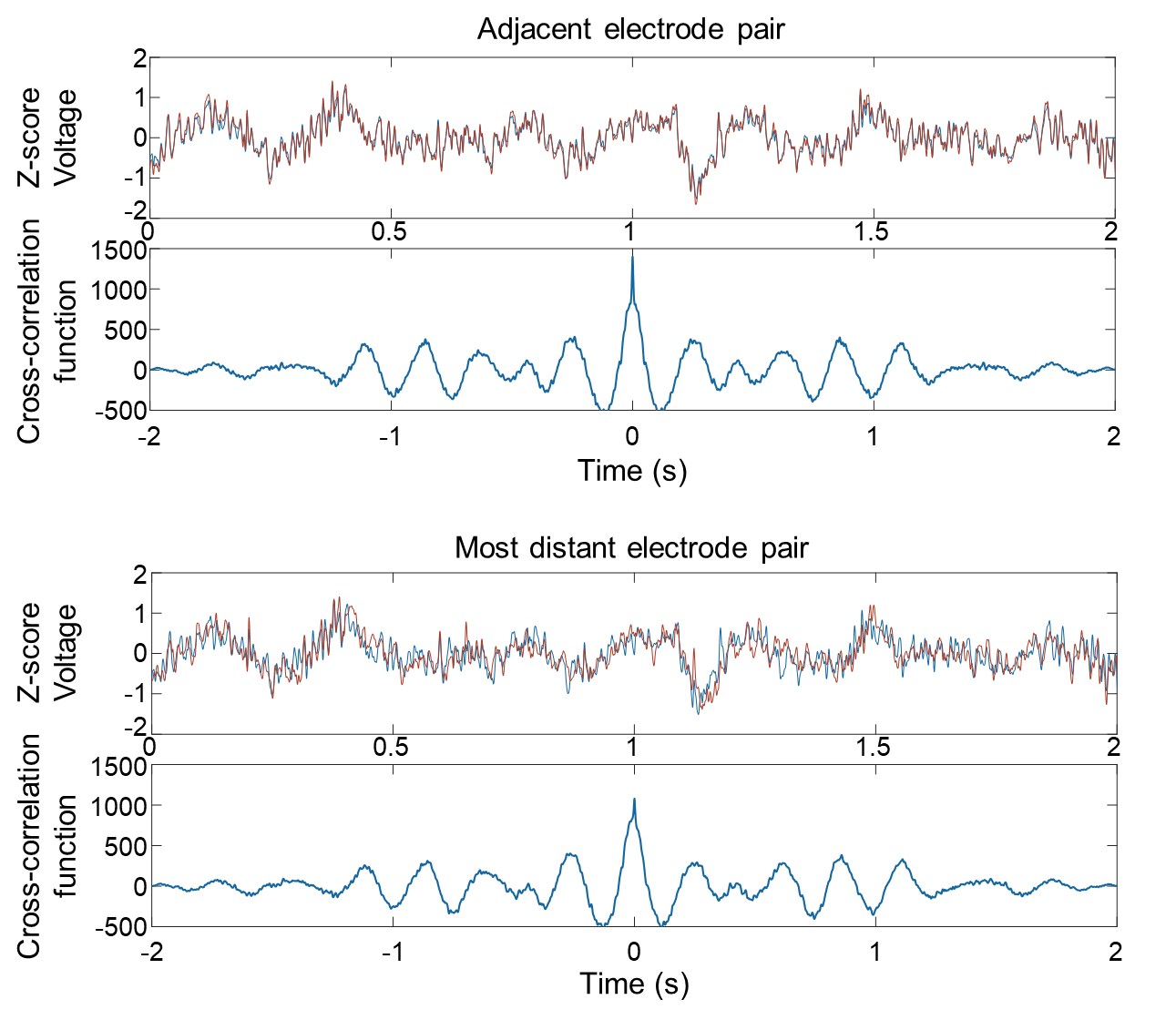


**Figure S20.** Example comparison of time-domain waveforms and their cross-correlation functions for adjacent and most distant electrode channel pairs. The μECoG recordings acquired by these two electrode pairs hardly differed in phase and had the same trend over longer time scales, reflecting the pattern of ECoG spatial spread. The peak of the cross-correlation function exhibits a distinct difference in magnitude, reflecting the relationship between the distance between in-pair electrodes and the similarity of the signals.

**
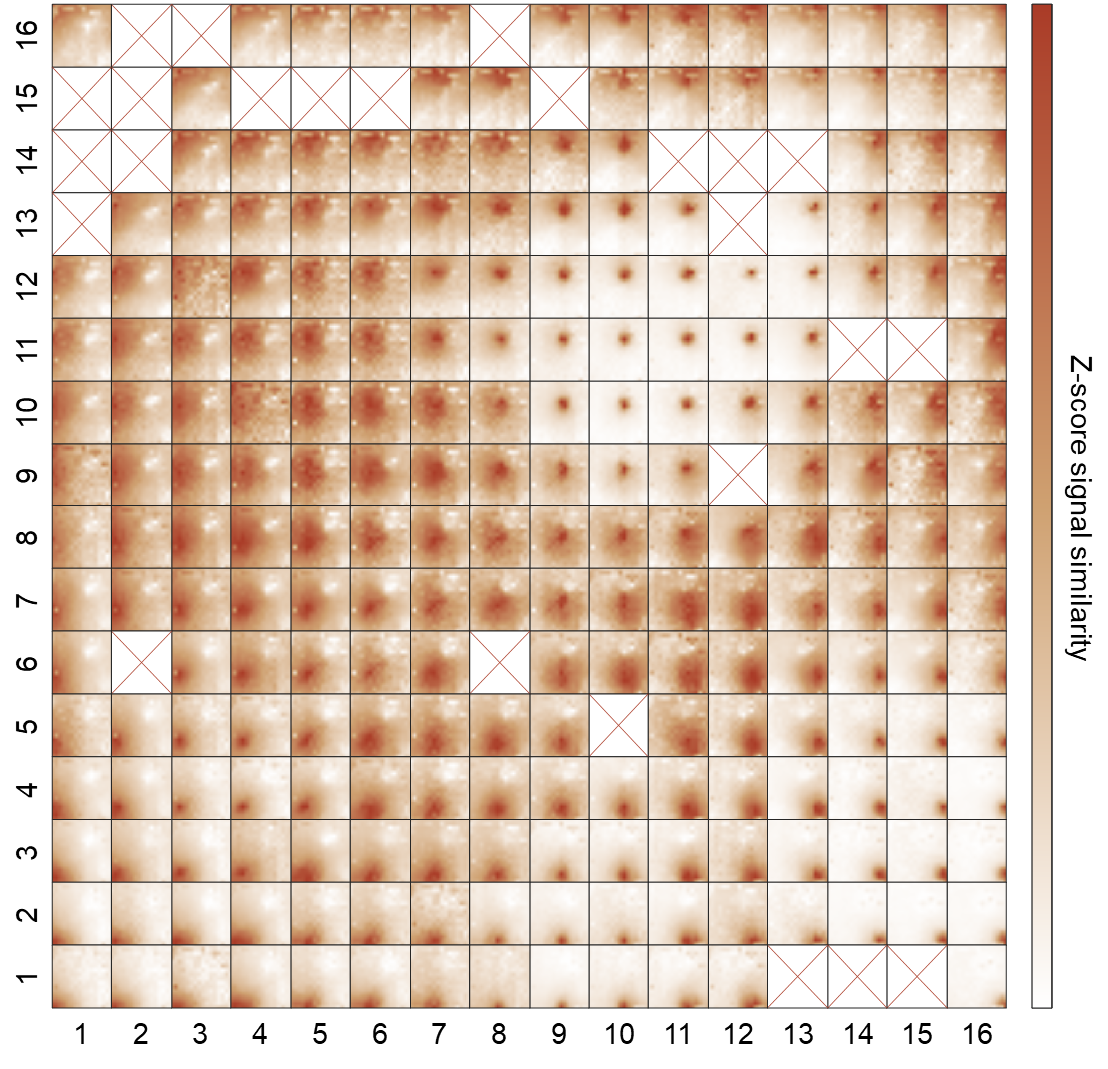
**

**Figure S21.** Spatial distribution of inter-electrode signal similarity. Each block represents the similarity of μECoG recordings between the electrode channel and others. The inter-electrode signal similarity has a clear specificity in spatial distribution. Combined with the results of the analysis of electrode contribution, it could be found that the μECoG recordings of high contribution electrode channels have a more pronounced spatial specificity.


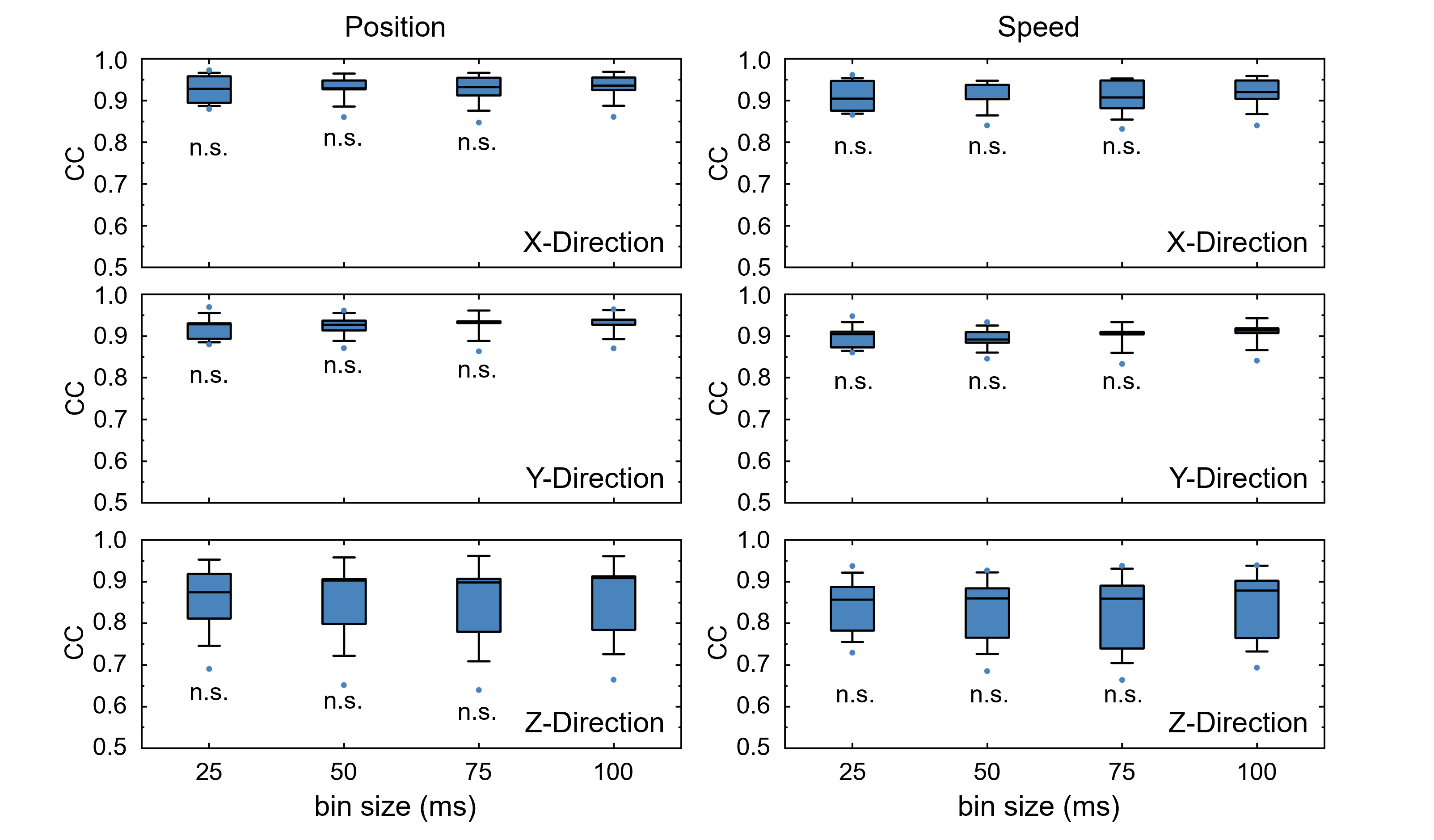


**Figure S22.** Decoding performance with varying time windows. In the box plot, the boxes represent the IQR, with an internal line marking the median. The whiskers extend to cover the range of mean ± standard deviation (Wilcoxon signed-rank tests were conducted for significance. n.s., p > 0.05, n = 5).


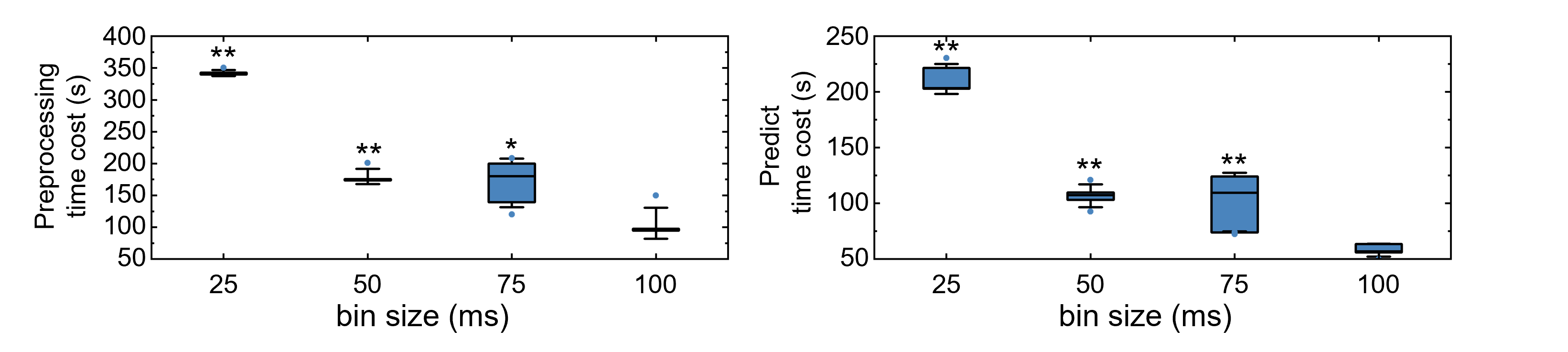


**Figure S23.** Time cost of preprocessing and predict with varying time windows. The time window of 100 ms achieve high decoding accuracy and low preprocessing time cost. In the box plot, the boxes represent the IQR, with an internal line marking the median. The whiskers extend to cover the range of mean ± standard deviation (Wilcoxon signed-rank tests were conducted for significance. n.s., p > 0.05, *p < 0.05, **p < 0.01, n = 5).


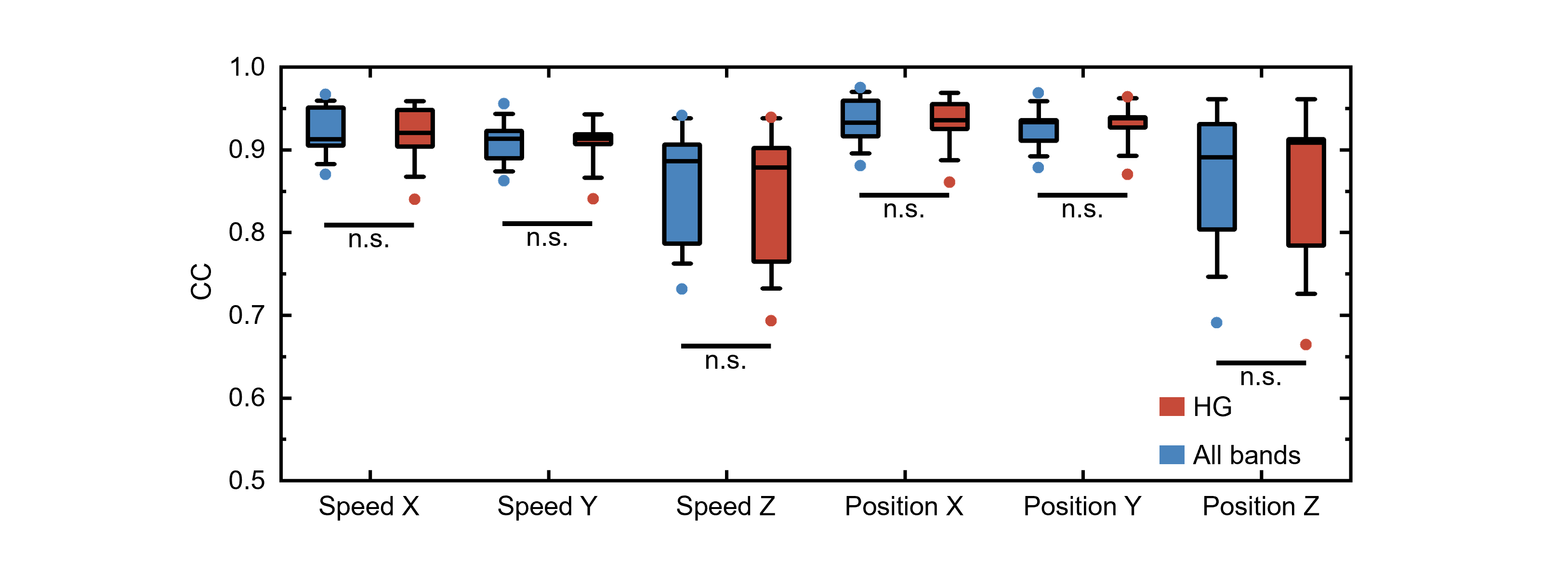


**Figure S24.** Comparative decoding performance using HG band versus multiple frequency bands (delta, theta, alpha, beta, gamma and HG) PSD. In the box plot, the boxes represent the IQR, with an internal line marking the median. The whiskers extend to cover the range of mean ± standard deviation (Wilcoxon signed-rank tests were conducted for significance. n.s., p > 0.05, n = 5).


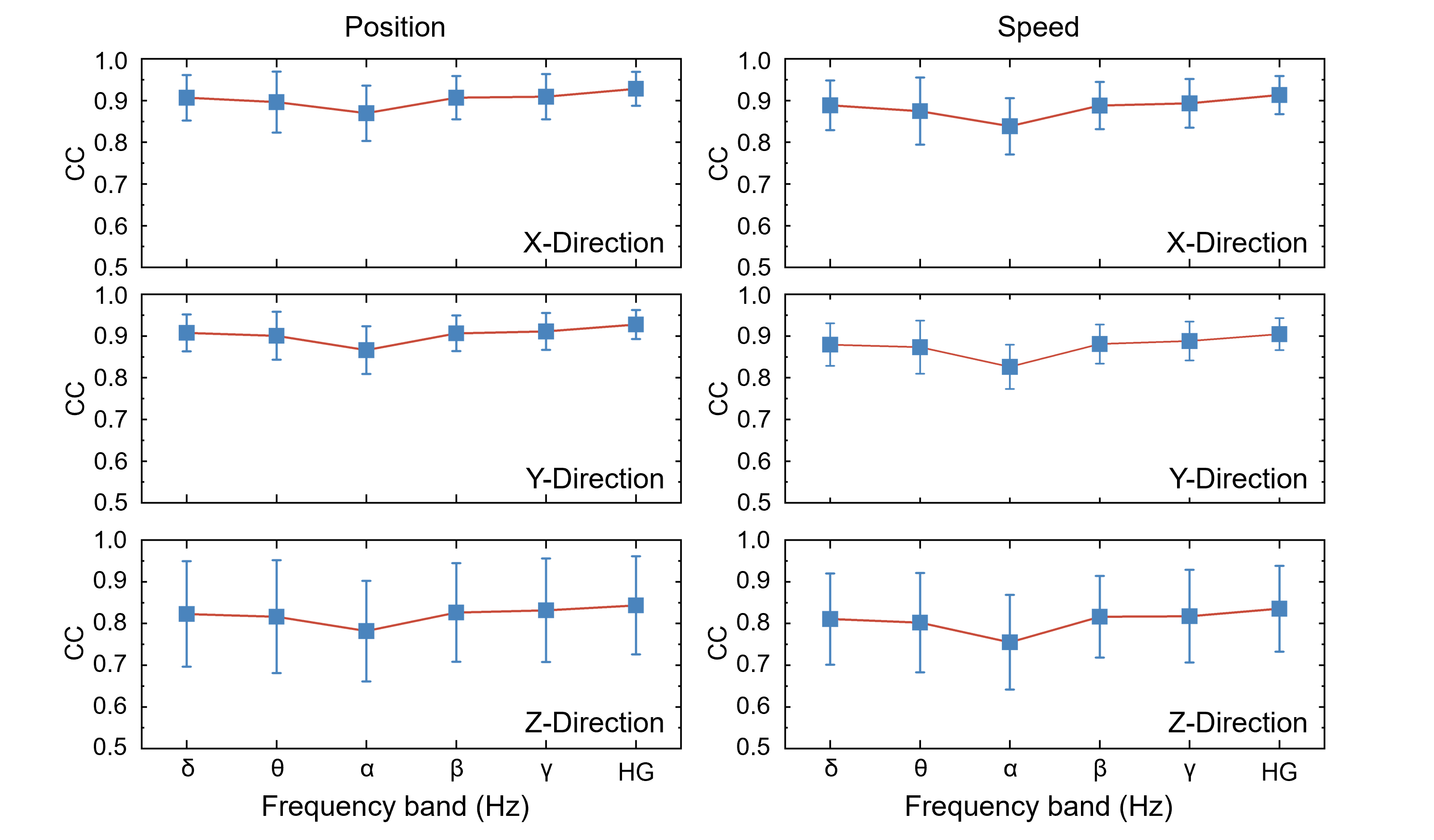


**Figure S25.** Motor decoding accuracy across isolated neural frequency bands. HG band features achive the highest decoding performance. In the point-line plot, square markers represent means, and error bars denote the range of mean ± standard deviation.


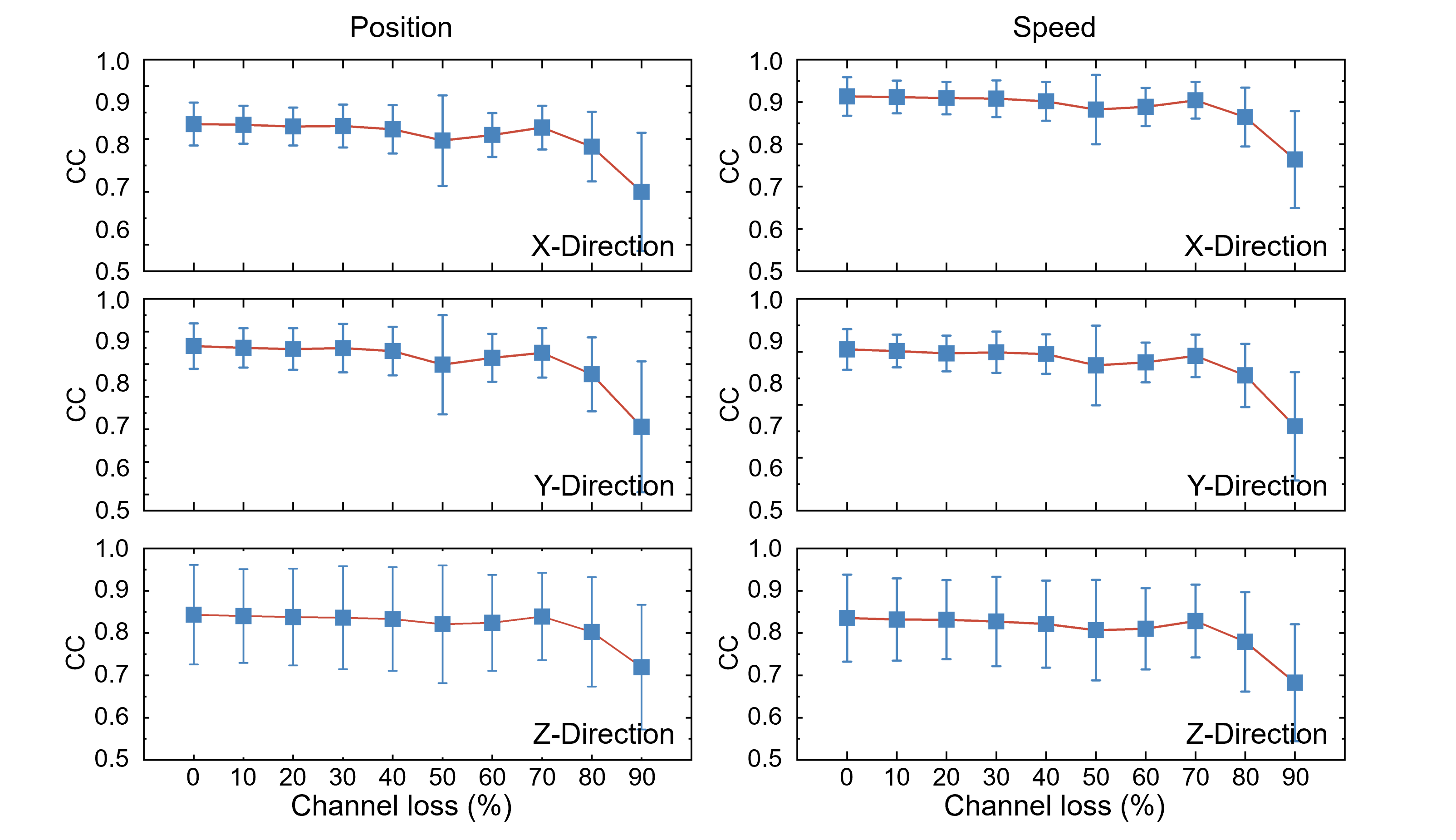


**Figure S26.** Decoding performance degradation under progressive exclusion of electrode channels. In the point-line plot, square markers represent means, and error bars denote the range of mean ± standard deviation.


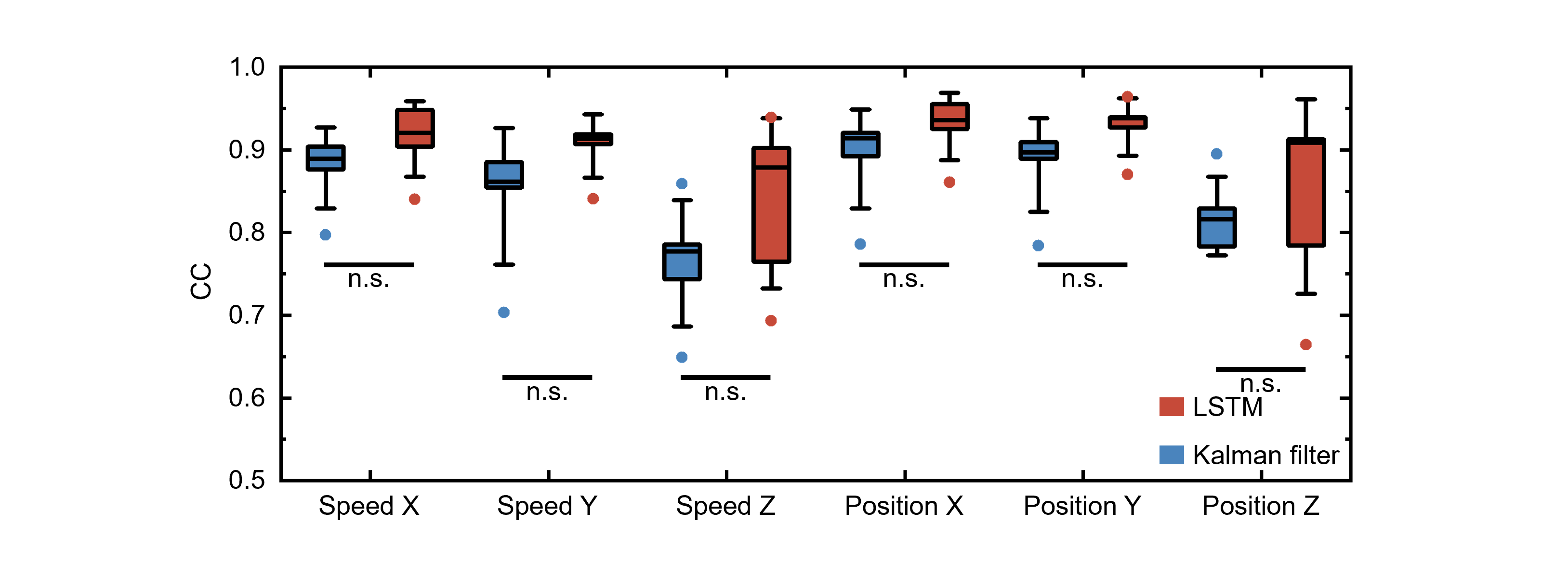


**Figure 27.** Performance comparison of the position-velocity Kalman filter and the LSTM decoders for motor decoding. Motor decoding was performed using position-velocity Kalman filter and LSTM decoders, respectively, and the Pearson correlation coefficient between the predicted and actual kinematic features was calculated as a measurement of decoding accuracy. In the box plot, the boxes represent the interquartile ranges (IQR), with an internal line marking the median. The whiskers extend to cover the range of mean ± standard deviation (Wilcoxon signed-rank tests were conducted for significance. n.s., p > 0.05, n = 5).

**Video S1.** Real-time multi-joint motor decoding in the canine model. Combining motion capture technology and real-time multi-modal monitoring, real-time motor decoding for multi-joint kinematic features is achieved with high accuracy.

**Video S2.** Intraoperative Ping-Pong game controlled by the μECoG BCI. After the model training phase of seven minutes, the Ping-Pong game could be controlled by ECoG recordings of the patient through real-time one dimension motor decoding by our μECoG BCI intraoperatively**.**

**Video S3.** Intraoperative Snake game controlled by the μECoG BCI. After the model training phase of seven minutes, the Snake game could be controlled by ECoG recordings of the patient through real-time two-dimension motor decoding by our μECoG BCI intraoperatively.

**Video S4.** Multiple applications through motor imagery decoding**.** After mastering the use of μECoG BCI to control the cursor, the participant was able to perform a variety of complex applications, including video games, intelligent wheelchair, and smart home controlling
